# Therapeutic effect and safety of stem cell therapy for chronic liver disease: a systematic review and meta-analysis of randomized controlled trials

**DOI:** 10.1186/s13287-020-01935-w

**Published:** 2020-09-25

**Authors:** Guang-Peng Zhou, Yi-Zhou Jiang, Li-Ying Sun, Zhi-Jun Zhu

**Affiliations:** 1 Liver Transplantation Center, National Clinical Research Center for Digestive Diseases, Beijing Friendship Hospital, Capital Medical University, No. 95 Yong-an Road, Xi-Cheng District, Beijing, 100050 China; 2 Clinical Center for Pediatric Liver Transplantation, Capital Medical University, No. 95 Yong-an Road, Xi-Cheng District, Beijing, 100050 China; 3 Intensive Care Unit, Beijing Friendship Hospital, Capital Medical University, Beijing, 100050 China

**Keywords:** Stem cell therapy, Liver disease, Cell transplantation, Acute-on-chronic liver failure

## Abstract

**Background:**

Stem cell therapy is becoming an emerging therapeutic option for chronic liver disease (CLD). However, whether stem cell therapy is more effective than conventional treatment remains questionable. We performed a large-scale meta-analysis of randomized controlled trials (RCTs) to evaluate the therapeutic effects and safety of stem cell therapy for CLD.

**Methods:**

We systematically searched MEDLINE, EMBASE, the Cochrane Central Register of Controlled Trials (CENTRAL), and ClinicalTrials.gov databases for the period from inception through March 16, 2020. Primary outcomes were all-cause mortality and adverse events related to stem cell therapy. Secondary outcomes included the model for end-stage liver disease score, total bilirubin, albumin, alanine aminotransferase, prothrombin activity, and international normalized ratio. The standardized mean difference (SMD) and odds ratio (OR) with 95% confidence interval (CI) were calculated using a random-effects model.

**Results:**

Twenty-four RCTs were included and the majority of these studies showed a high risk of bias. The meta-analysis indicated that compared with conventional treatment, stem cell therapy was associated with improved survival and liver function including the model of end-stage liver disease score, total bilirubin, and albumin levels. However, it had no obvious beneficial effects on alanine aminotransferase level, prothrombin activity, and international normalized ratio. Subgroup analyses showed stem cell therapy conferred a short-term survival benefit for patients with acute-on-chronic liver failure (ACLF), a single injection was more effective than multiple injections, hepatic arterial infusion was more effective than intravenous infusion, and bone marrow-derived stem cells were more effective than those derived from the umbilical cord. Thirteen trials reported adverse events related to stem cell therapy, but no serious adverse events were reported.

**Conclusions:**

Stem cell therapy is a safe and effective therapeutic option for CLD, while patients with ACLF benefit the most in terms of improved short-term survival. A single injection administration of bone marrow-derived stem cells via the hepatic artery has superior therapeutic effects.

## Background

Chronic liver disease (CLD), mainly those arising from hepatitis viral infection, toxic injury, alcohol abuse, metabolic disorders, or genetic defect, is an important global health concern. In China alone, it was estimated that more than 400 million people suffered CLD, primarily viral hepatitis, metabolic associated fatty liver disease, and alcoholic liver disease [1]. Given the natural history of CLD, these patients are at high risk of progressing to advanced fibrosis, cirrhosis, and cirrhosis-related complications including acute-on-chronic liver failure (ACLF) and hepatocellular carcinoma. Cirrhosis and liver cancer ranked the 11st and 16th among the leading causes of death worldwide in 2016, respectively, representing a death toll of more than 2 million [2]. Currently, liver transplantation (LT) is the ultimate curative treatment for end-stage liver disease. However, limited organ availability, high costs, transplant-associated complications, and lifelong immunological side effects preclude many patients from benefiting from LT [3, 4]. Therefore, people have been seeking alternative therapeutic strategies to LT.

Stem cell therapy is becoming an emerging therapeutic option for CLD with great potential, because it is a less invasive curative with potentially equal effect compared to LT [5, 6]. Although a growing number of clinical researches, ranging from early proof-of-concept studies to randomized controlled trials (RCTs), have been carried out to explore the safety and efficacy of stem cell therapy in a range of different settings of liver diseases, whether stem cell therapy is associated with better therapeutic effects than conventional treatment remains unknown and its safety profile as well [7, 8]. More importantly, regarding cell source (autologous or allogeneic; bone marrow or umbilical cord blood), administration dose, infusion route (intrahepatic, intrasplenic, or intravenous), and delivery frequency (singular or multiple), no standardized protocols have been published to date, although these factors are undoubtedly the leading ones among those influencing the therapeutic effects of stem cell therapy, and they can even cause a series of side effects [7, 9, 10].

Previous systematic reviews pooled analysis of both RCTs and non-RCTs [11–13], but studies of different designs should not be analyzed in unification. Furthermore, none of them has included all relevant randomized trials, while RCTs are assessed as the best corroboration of the efficacy of new treatments in evidence-based medicine. Thus, previously published systematic reviews only have limited power to determine whether patients with CLD can benefit from stem cell therapy. Therefore, we conducted a systematic review and meta-analysis of all currently available RCTs to provide a more comprehensive and quantitative understanding of the therapeutic effects and safety of stem cell therapy for treating CLD.

## Methods

This systematic review and meta-analysis has been registered in PROSPERO (CRD42020175317). We followed the recommendations from the Cochrane Collaboration for systematic review and meta-analysis of RCTs and reported following the Preferred Reporting Items for Systematic Reviews and Meta-analyses statement [14].

### Search strategy

We searched RCTs involving CLD patients treated with implantation of all kinds of stem cells from electronic medical databases including MEDLINE (PubMed), Ovid EMBASE, the Cochrane Central Register of Controlled Trials (CENTRAL), and ClinicalTrials.gov from initial period to March 16, 2020. Key searching terms were “liver disease,” “stem cells,” “stem cell transplantation,” and “randomized controlled trial.” MeSH terms and free-text terms, as well as variation of root words, were combined within each database. No language restrictions were applied during the searches. The reference list of the eligible articles and relevant review articles were also checked to identify additional studies. The detailed search strategies are outlined in Additional file 1: Table S1.

### Study selection

Two reviewers (G-PZ, Y-ZJ) independently screened the titles and abstracts of retrieved publications. We retrieved the full-text articles of the studies that were deemed potentially eligible for a review as a whole. Any disagreements were resolved through discussion with a third reviewer (L-YS). The inclusion criteria were (1) RCTs, (2) patients diagnosed with CLD, (3) patients in the experimental group received stem cell therapy and patients in the control group were treated with conventional treatment, and (4) availability of clinical outcomes. Primary outcomes were all-cause mortality and adverse events related to stem cell therapy. Secondary outcomes included the model for end-stage liver disease (MELD) score, liver function parameters (total bilirubin (TBIL), albumin (ALB), and alanine aminotransferase (ALT)), and coagulation function (prothrombin activity (PTA) and international normalized ratio (INR)). Studies were excluded if (1) they were animal-based, review articles, or case reports or (2) their full-text or adequate information was not available. When duplicate reports from the same study were identified, only the one with more information was included.

### Data extraction

Two authors (G-PZ, Y-ZJ) extracted data from included studies independently, and disagreements were resolved through a discussion with a third reviewer (L-YS). The following information was extracted from the included studies using a predefined data form: study characteristics (first author, year of publication, country, study design, enrollment period, number of participants in the experimental and control groups, and follow-up duration), patient characteristics (age, sex, and liver disease type), stem cells (number, type, delivery route, and frequency of administration), and outcome measures.

### Quality assessment

The risk of bias for each included study was independently assessed by two authors (G-PZ, Y-ZJ) using the Cochrane Collaboration’s Risk of Bias tool [15]. The evaluation domains included selection bias (allocation sequence generation, allocation concealment), performance bias (blinding of participants and personnel), detection bias (blinding of outcome assessment), attrition bias (incomplete outcome data), reporting bias (selective outcome reporting), and other bias. For each domain, studies were judged as low, high, or unclear risk of bias according to the Cochrane Handbook.

### Statistical analysis

By using a random-effects model, continuous and dichotomous outcome variables were calculated as standardized mean difference (SMD) and odds ratio (OR) with 95% confidence interval (CI), respectively. Heterogeneity between studies was assessed using Cochran’s *Q* test and *I*^*2*^ statistic. In case of substantial heterogeneity (*I*^*2*^ > 50%), a sensitivity analysis with omission of one study at a time was conducted. To explore the potential influence factors of stem cell therapy for treating CLD, pre-planned subgroup analyses based on liver disease type (ACLF and CLD without ACLF), cell type [bone marrow-derived mesenchymal stem cells (BM-MSCs), bone marrow-derived mononuclear stem cells (BM-MNCs), or umbilical cord-derived mesenchymal stem cells (UC-MSCs)], delivery route (peripheral intravenous infusion and hepatic arterial infusion), and frequency of administration (single injection and multiple injections) were performed. Where sufficient studies were available (the number of included studies ≥ 10), publication bias was evaluated based on the funnel plot, Egger’s test for continuous endpoints and Harbord’s test for dichotomous endpoints [16, 17]. If the funnel plot was asymmetrical, contour-enhanced funnel plots combined with trim and fill analysis was conducted to explore the source of publication bias [18, 19]. A *P* value of less than 0.05 was considered statistically significant. All statistical analyses were performed using the Review Manager software (version 5.3) and STATA 14 software (Stata Corp).

## Results

### Study selection

**Fig. 1:**
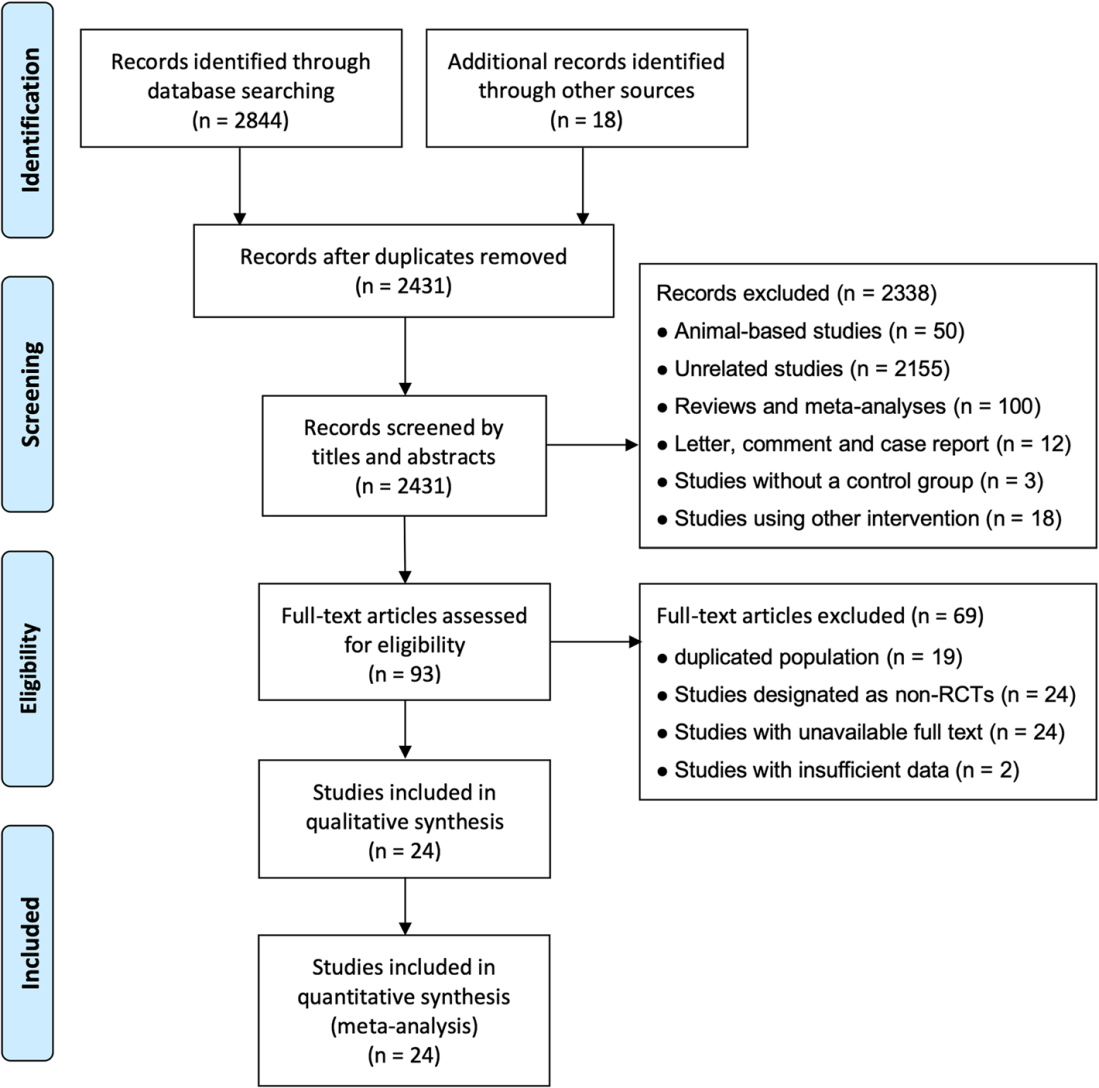
Flow diagram of the selection of studies

We identified a total of 2862 potentially eligible articles by searching the four databases and the reference lists of retrieved articles and relevant reviews, of which 431 were excluded due to duplication. After the title and abstract review, 2338 articles were further excluded, with 93 potentially relevant articles left. After a detailed assessment of the full texts, 69 papers were further excluded. Finally, 24 studies [20–43] were included in the present meta-analysis. The flow diagram of the selection of studies is listed in Fig. 1.

### Study characteristics

The characteristics of the 24 included studies are presented in Table 1. These studies were published between 2010 and 2019 from Brazil (*n* = 1), China (*n* = 13), Egypt (*n* = 4), Iran (*n* = 3), South Korea (*n* = 1), Switzerland (*n* = 1), and the UK (*n* = 1). A total of 1359 patients were included, with 746 patients receiving stem cell therapy and 613 patients undergoing conventional treatment. The studies included patients with liver fibrosis (*n* = 1), liver cirrhosis (*n* = 17), and liver failure (*n* = 6). Stem cells were derived from the bone marrow (BM-MSCs; *n* = 8 and BM-MNCs; *n* = 6), umbilical cord (UC-MSCs; *n* = 8), and peripheral blood (PBSCs; *n* = 2), 15 of which involve autologous transplants while the remaining ones involve allogeneic transplants. Stem cells were delivered into the liver through the peripheral vein (*n* = 12), hepatic artery (*n* = 7), portal vein (*n* = 2), or multiple routes (*n* = 3). A single cell injection was adopted in 11 studies, multiple cell injections in 11 studies, and both (single and multiple injections) in 2 studies.

**Table 1:** Characteristics of the included randomized controlled studies

Study	Country	Patient population	Enrollment period	Sample size (Exp/Con)	Male (Exp/Con)	Average age (Exp/Con)	Cell type	Delivery route	Times of injection	Number of stem cells	Follow-up period (weeks)
Lyra AC, 2010 [20]	Brazil	Advanced chronic liver disease	2006.1–2006.4	15/15	N/A	56.7/50.0	Autologous BM-MNCs	Hepatic artery	Single	3.0 (0.88–11.2) × 10^8^	48
Salama H, 2010 [21]	Egypt	End-stage liver cirrhosis	2008.6–2009.5	90/50	78/39	50.3/50.9	Autologous BM-HSCs	Portal vein	Single	0.5 × 10^8^	24
Amer ME, 2011 [22]	Egypt	HCV-related end-stage liver failure	2008.10–2009.6	20/20	16/17	50.5/50.0	Autologous BM-MSCs	Intrasplenic injection/portal vein	Single	2.0 × 10^8^	24
Lin H, 2012 [23]	China	Decompensated liver cirrhosis	2009.1–2010.1	38/16	34/15	47/48*	Allogeneic UC-MSCs	Peripheral vein	Multiple	(0.5–1.0) × 10^6^/kg	48
Shi M, 2012 [24]	China	HBV-related ACLF	2009.3–2010.9	24/19	20/15	40/45*	Allogeneic UC-MSCs	Peripheral vein	Multiple	0.5 × 10^6^/kg	72
Zhang YF, 2012 [25]	China	Decompensated liver cirrhosis	2009.3–2010.12	12/18	8/13	48.6/49.9	Allogeneic UC-MSCs	Hepatic artery	Single	≥ 2.0 × 10^7^	12
Zhang Z, 2012 [26]	China	HBV-related decompensated liver cirrhosis	N/A	30/15	26/14	48/47*	Allogeneic UC-MSCs	Peripheral vein	Multiple	0.5 × 10^6^/kg	48
Mohamadnejad M, 2013 [27]	Iran	Decompensated liver cirrhosis	2007.7–2010.8	14/11	7/6	43.1/34.6	Autologous BM-MSCs	Peripheral vein	Single	(1.2–2.95) × 10^8^	48
Spar L, 2013 [28]	Switzerland	Decompensated alcoholic liver disease	2008.2–2011.3	28/30	24/20	54/56*	Autologous BM-MNCs	Hepatic artery	Single	(0.47 ± 0.15) × 10^8^/kg	12
Wang QC, 2013 [29]	China	Decompensated liver cirrhosis and chronic liver failure	2011.11–2010.5	9/9	14	50.7	Allogeneic UC-MSCs	Peripheral vein	Multiple	(1.2–6.2) × 10^7^/mL	4
Salama H, 2014 [30]	Egypt	HCV-related end-stage liver disease	2010.6–2011.10	20/20	17/16	50.3/50.9	Autologous BM-MSCs	Peripheral vein	Single	1 × 10^6^/kg	26
Xu L, 2014 [31]	China	HBV-related liver cirrhosis	2012.3–2012.12	20/19	13/11	44/45	Autologous BM-MSCs	Hepatic artery	Single	(0.75 ± 0.5) × 10^6^	24
Deng QZ, 2015 [32]	China	HBV-related decompensated liver cirrhosis	2011.7–2013.12	33/35	20/12	49.5/50.2	Autologous PBSCs	Hepatic artery	Single	(2–4) × 10^7^	48
Li YY, 2015 [33]	China	HBV-related ACLF	2009.10–2015.5	31/27	28/24	41.6/43.1	Allogeneic UC-MSCs	Peripheral vein	Multiple	(0.5–1.0) × 10^6^/kg	48
Zekri AR, 2015 [34]	Egypt	HCV-related liver cirrhosis	2010.5–2012.5	60/30	51/26	50.3/49.4	Autologous BM-CD34+/CD133+ cells	Portal vein/peripheral vein	Single/multiple	1 × 10^6^/kg	48
Mohamadnejad M, 2016 [35]	Iran	Decompensated liver cirrhosis	2010.3–2012.6	10/9	7/5	43.9/46.2	Autologous BM-MNCs	Portal vein	Multiple	(7.62 ± 5.53) × 10^6^ (9.17 ± 5.24) × 10^6^	48
Suk KT, 2016 [36]	South Korea	Alcoholic liver cirrhosis	2013.1–2015.11	37/18	32/17	53.8/53.7	Autologous BM-MSCs	Hepatic artery	Single/multiple	5 × 10^7^	48
Fang XQ, 2017 [37]	China	HBV-related decompensated liver cirrhosis	2013.1–2016.5	59/59	43/41	51.8/50.4	Allogeneic UC-MSCs	Hepatic artery/peripheral vein	Multiple	(4.0–4.5) × 10^8^	52
Lin BL, 2017 [38]	China	HBV-related ACLF	2010.10–2013.4	56/54	51/53	40.0/42.8	Allogeneic BM-MSCs	Peripheral vein	Multiple	(1.0–10) × 10^5^/kg	24
Wu YZ, 2017 [39]	China	HBV-related decompensated liver cirrhosis	2014.3–2016.2	42/42	25/24	49/50	Autologous BM-MSCs	Hepatic artery	Single	1 × 10^6^/kg	48
Zhang D, 2017 [40]	China	Liver fibrosis	2012.1–2015.1	30/30	16/17	31.0/32.1	Autologous BM-MSCs	Peripheral vein	Multiple	3 × 10^6^/mL	12
Newsome PN, 2018 [41]	UK	Compensated liver cirrhosis	2010.5–2015.2	28/27	22/13	56.5/52.0	Autologous PBSCs	Peripheral vein	Multiple	0.6 × 10^6^/kg	48
Esmaeilzadeh A, 2019 [42]	Iran	Decompensated liver cirrhosis	2014.9–2015.6	10/10	9/8	46.0/45.2	Autologous BM-MNC	Peripheral vein	Single	(2.15–12.3) × 10^6^/kg	24
Xu WX, 2019 [43]	China	HBV-related ACLF	2012.1–2017.9	30/30	29/28	40.7/45.0	Allogeneic UC-MSCs	Peripheral vein	Multiple	1.0 × 10^5^/kg	48

*Abbreviations*: *Con* control group (conventional treatment), *Exp* experimental group (stem cell therapy), *BM* bone marrow, *MSC* mesenchymal stem cell, *HSC* hematopoietic stem cell, *UC* umbilical cord, *MNC* mononuclear cell, *N/A* not available, *PBSC* peripheral blood stem cell, *HBV* hepatitis B virus, *HCV* hepatitis C virus, *ACLF* acute-on-chronic liver failure

*Median value

### Risk of bias of the included studies

**Fig. 2:**
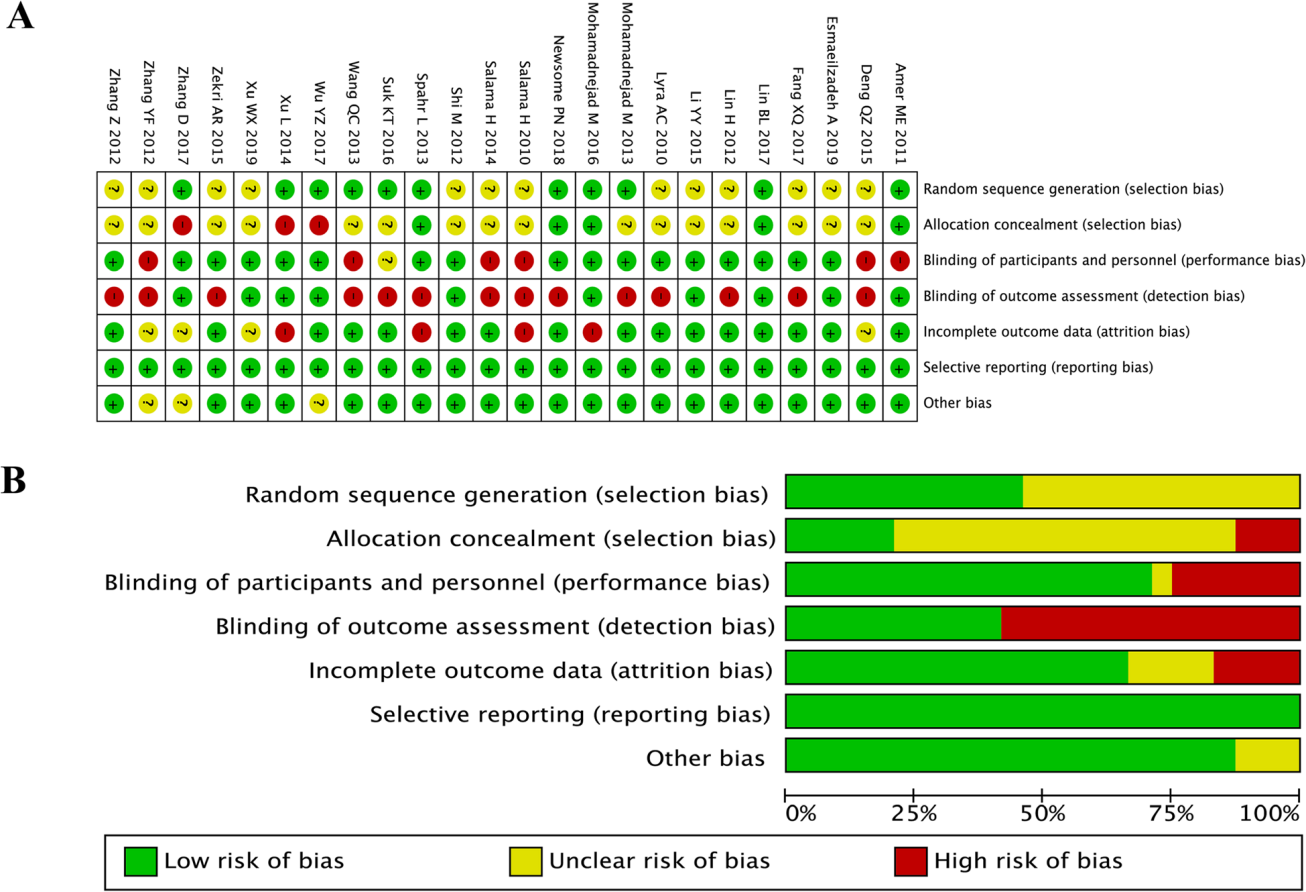
**a** Risk of bias summary: review of authors’ judgments about each risk of bias item for each included study. **b** Risk of bias graph: overview of authors’ judgments about each risk of bias item presented as percentages across all included studies

The majority of the included studies showed a high risk of bias, which mainly resulted from the lack of allocation concealment, absent blinding, and incomplete outcome data. Three studies [25, 39, 40] were considered to have an unclear risk of other bias because it is unclear whether there is free of for-profit bias. Further details are presented in Fig. 2.

### Therapeutic safety and efficacy assessments

#### All-cause mortality

**Fig. 3:**
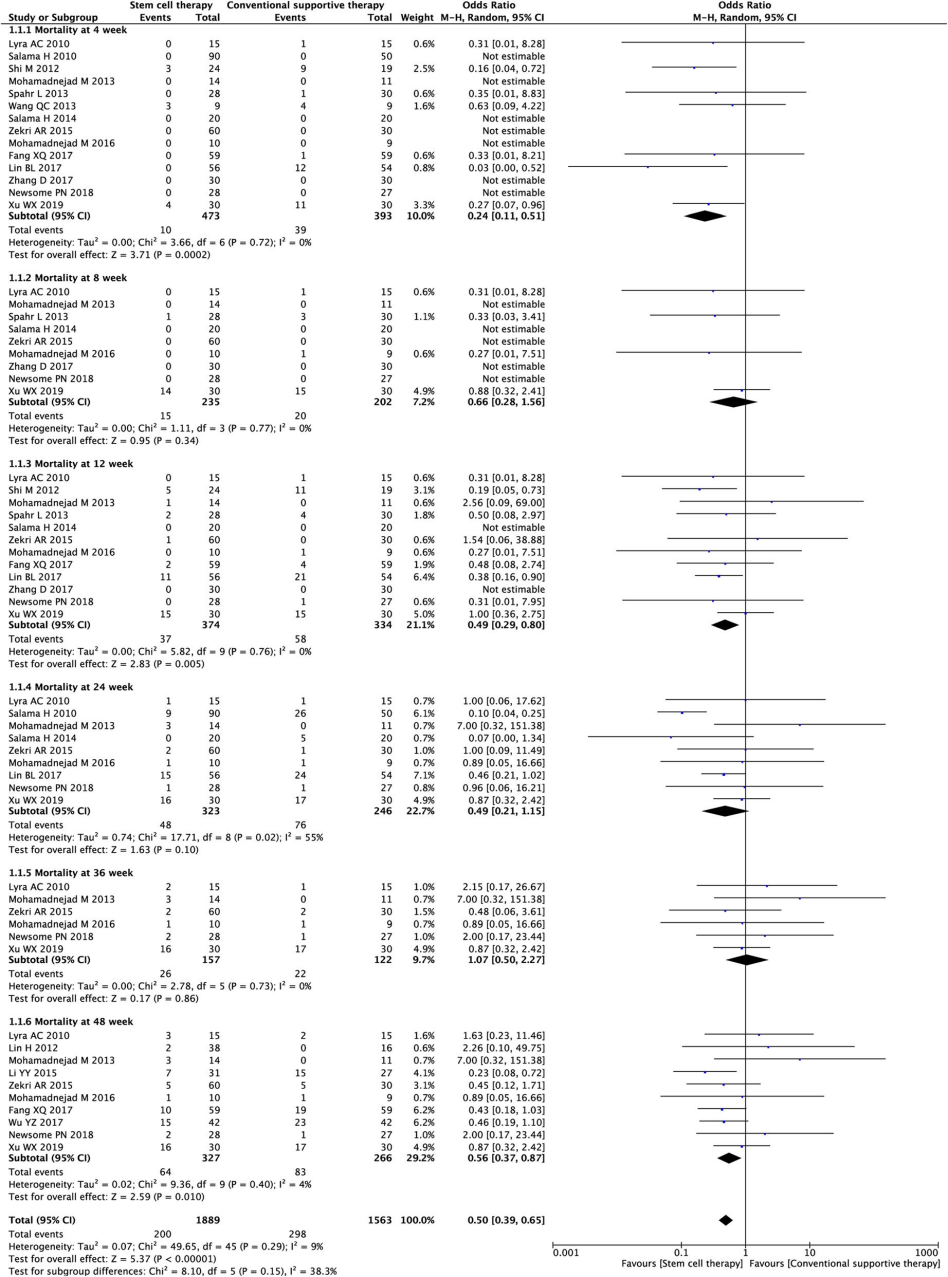
Forest plot of the comparison of the effect of stem cell therapy versus conventional treatment on all-cause mortality

Seventeen studies (3452 participants) were included in the analysis of all-cause mortality (Fig. 3). Compared with conventional treatment, stem cell therapy was associated with significantly lower all-cause mortality, as indicated by decreased all-cause mortality at week 4 (OR = 0.24, 95% CI 0.11 to 0.51; *P* = 0.0002), week 12 (OR = 0.49, 95% CI 0.29 to 0.80; *P* = 0.005), and week 48 (OR = 0.56, 95% CI 0.37 to 0.87; *P* = 0.01).

We found substantial heterogeneity at week 24 only (*I*^*2*^ = 55%). By excluding one study [21], sensitivity analyses showed the heterogeneity among the remaining studies was eliminated (Additional file 2: Table S2). Publication bias was evaluated at weeks 4, 12, and 48, and the funnel plot and Harbord’s test indicated evident publication bias at week 48 (Additional file 3: Fig. S1). Symmetrical contour-enhanced funnel plot combined with trim and fill analysis suggested the asymmetry in the funnel plot was partly attributed to publication bias (Additional file 4: Fig. S2).

#### MELD score

**Fig. 4:**
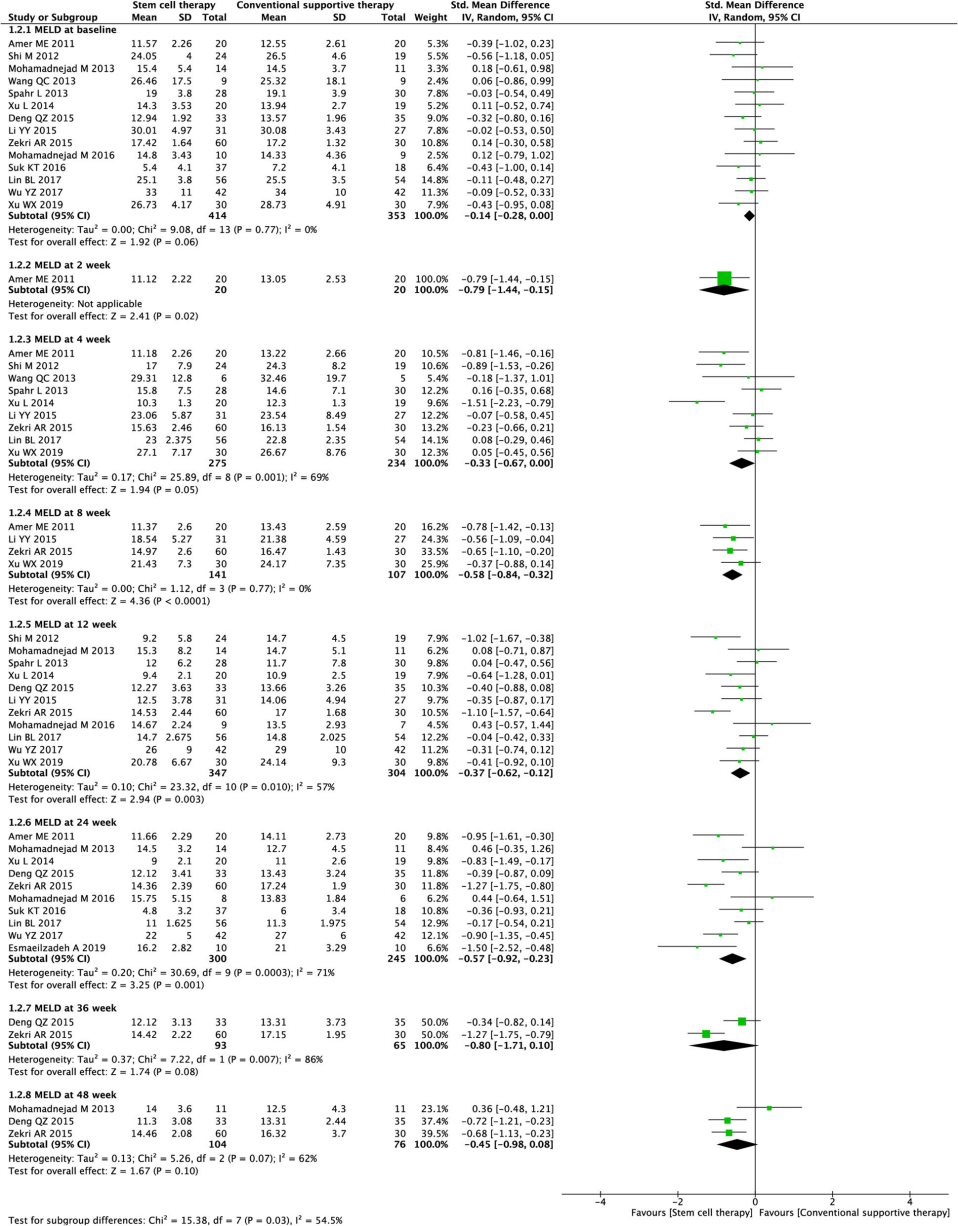
Forest plot of the comparison of the effect of stem cell therapy versus conventional treatment on the model of end-stage liver disease (MELD) score

Fifteen studies (3098 participants) were included in the analysis of MELD scores (Fig. 4). Before treatment, no significant difference was observed between the experimental and control groups (SMD = − 0.14, 95% CI − 0.28 to 0.00; *P* = 0.06). After treatment, stem cell therapy was associated with significantly lower MELD scores at week 2 (SMD = − 0.79, 95% CI − 1.44 to − 0.15; *P* = 0.02), week 8 (SMD = − 0.58, 95% CI − 0.84 to − 0.32; *P* < 0.0001), week 12 (SMD = − 0.37, 95% CI − 0.62 to − 0.12; *P* = 0.003), and week 24 (SMD = − 0.57, 95% CI − 0.92 to − 0.23; *P* = 0.001).

We found substantial heterogeneity at most of the time points (*I*^*2*^ = 57–86%). By excluding the results of Xu et al. [31] at week 4, Zekri et al. [34] at week 12, and Mohamadnejad et al. [27] at week 48, sensitivity analyses showed lowered heterogeneity among the remaining studies at each time point (Additional file 2: Table S2). Publication bias was evaluated at weeks 12 and 24, and the funnel plot and Egger’s test indicated no evident publication bias (Additional file 3 Fig. S1).

#### TBIL level

**Fig. 5:**
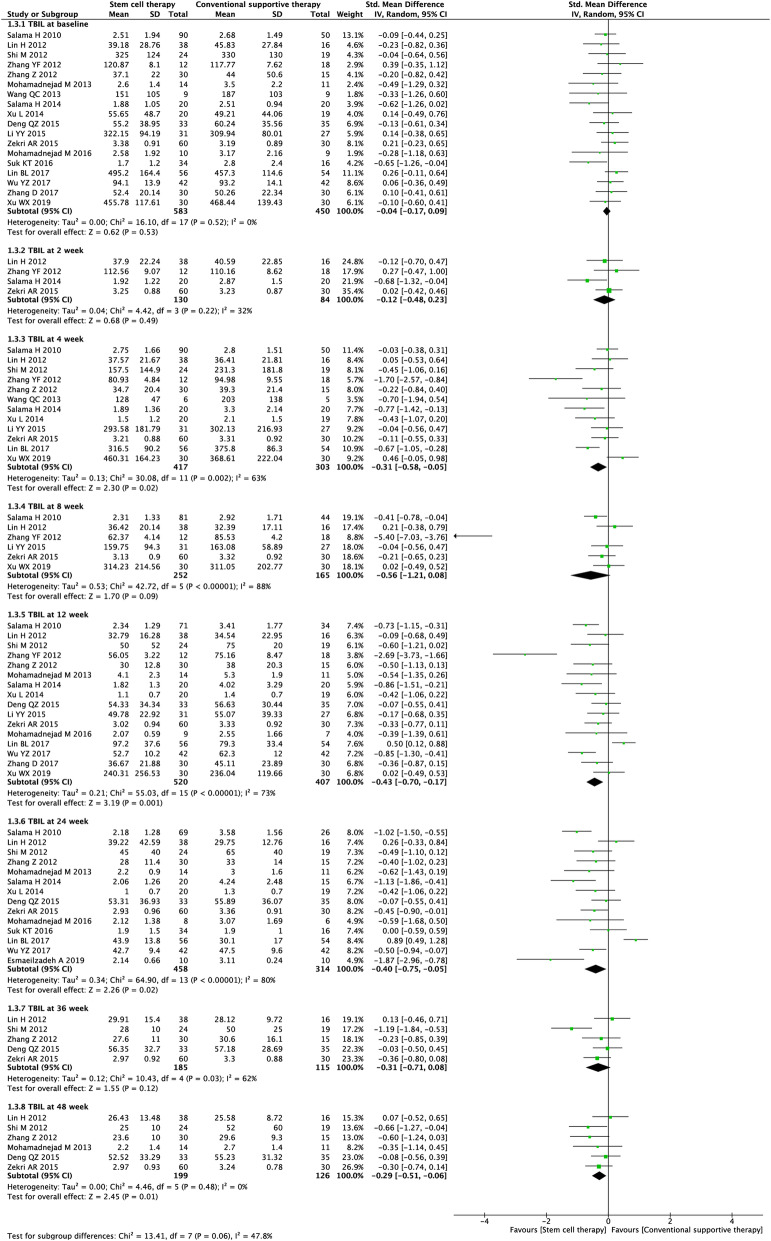
Forest plot of the comparison of the effect of stem cell therapy versus conventional treatment on total bilirubin (TBIL) level

Nineteen studies (4708 participants) were included in the analysis of the TBIL level (Fig. 5). Before treatment, no significant difference was observed between the experimental and control groups (SMD = − 0.04, 95% CI − 0.17 to 0.09; *P* = 0.53). After treatment, stem cell therapy was associated with significantly lower TBIL levels at week 4 (SMD = − 0.31, 95% CI − 0.58 to − 0.05; *P* = 0.02), week 12 (SMD = − 0.43, 95% CI − 0.70 to − 0.17; *P* = 0.001), week 24 (SMD = − 0.40, 95% CI − 0.75 to − 0.05; *P* = 0.02), and week 48 (SMD = − 0.29, 95% CI − 0.51 to − 0.06; *P* = 0.01).

We found substantial heterogeneity at most of the time points (*I*^*2*^ = 62–88%). By excluding the results of Zhang et al. [25] at weeks 4, 8, and 12, Lin et al. [38] at weeks 12 and 24, and Shi et al. [24] at week 36, sensitivity analyses showed lowered heterogeneity among the remaining studies at each time point (Additional file 2: Table S2). Publication bias was evaluated at weeks 4, 12, and 24, and the funnel plot and Egger’s test indicated evident publication bias in the TBIL level at week 12 (Additional file 1: Fig. S1). Symmetrical contour-enhanced funnel plot combined with trim and fill analysis suggested the asymmetry in the funnel plot was not caused by publication bias (Additional file 1: Fig. S2).

#### ALB level

**Fig. 6:**
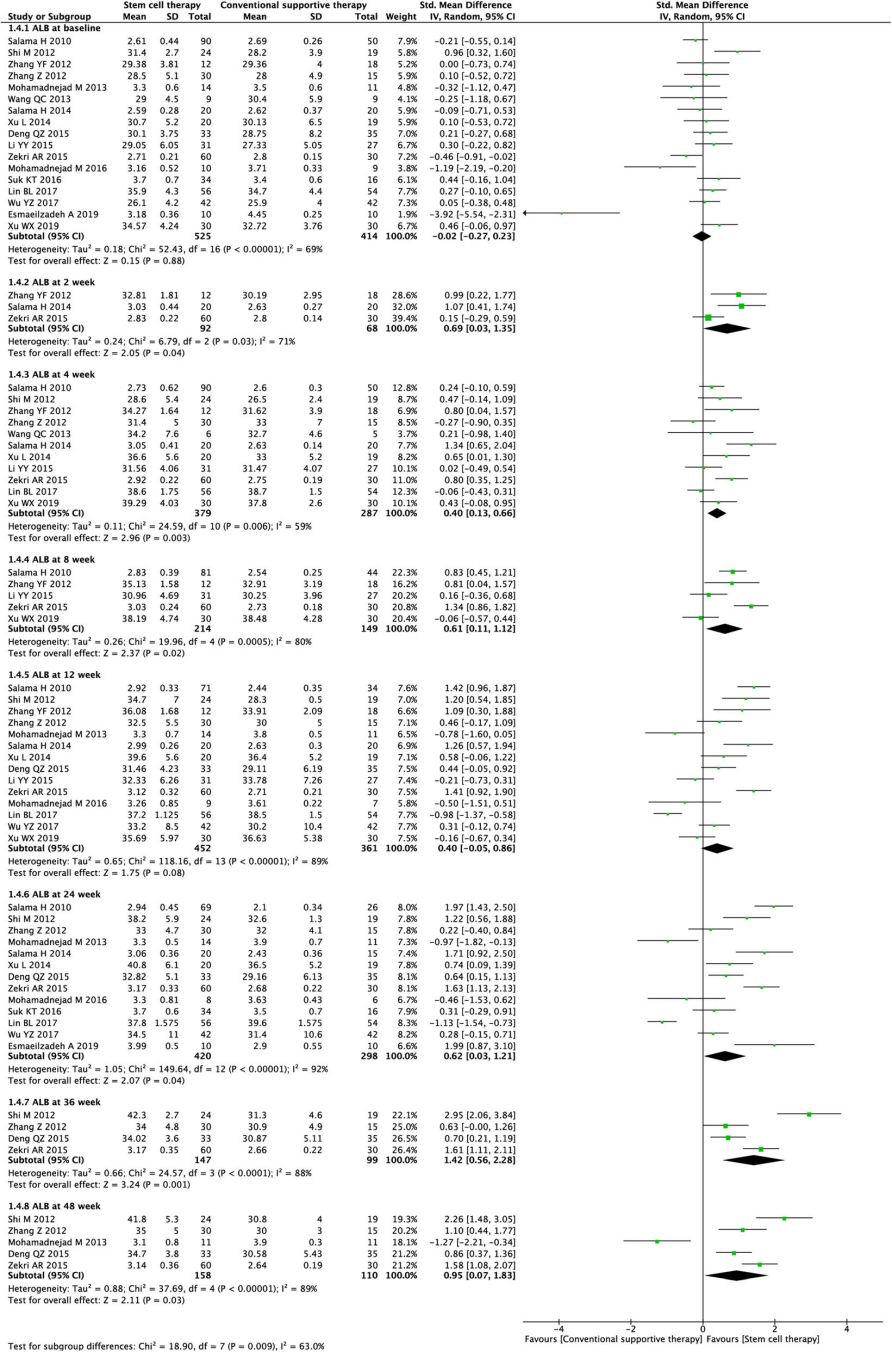
Forest plot of the comparison of the effect of stem cell therapy versus conventional treatment on albumin (ALB) level

Seventeen studies (4173 participants) were included in the analysis of the ALB level (Fig. 6). Before treatment, no significant difference was observed between the experimental and control groups (SMD = − 0.02, 95% CI − 0.27 to 0.23; *P* = 0.88). After treatment, stem cell therapy was associated with significantly increased ALB levels at week 2 (SMD = 0.69, 95% CI 0.03 to 1.35; *P* = 0.04), week 4 (SMD = 0.40, 95% CI 0.13 to 0.66; *P* = 0.003), week 8 (SMD = 0.61, 95% CI 0.11 to 1.12; *P* = 0.02), week 24 (SMD = 0.62, 95% CI 0.03 to 1.21; *P* = 0.04), week 36 (SMD = 1.42, 95% CI 0.56 to 2.28; *P* = 0.001), and week 48 (SMD = 0.95, 95% CI 0.07 to 1.83; *P* = 0.03).

We found substantial heterogeneity at all time points (*I*^*2*^ = 59–92%). By excluding the results of Zekri et al. [34] at week 2, Salama et al. [30] at week 4, and Mohamadnejad et al. [27] at week 48, sensitivity analyses showed lowered heterogeneity among the remaining studies at each time point (Additional file 2: Table S2). Publication bias was evaluated at weeks 4, 12, and 24, and the funnel plot and Egger’s test indicated no evident publication bias (Additional file 3 Fig. S1).

#### ALT level

**Fig. 7:**
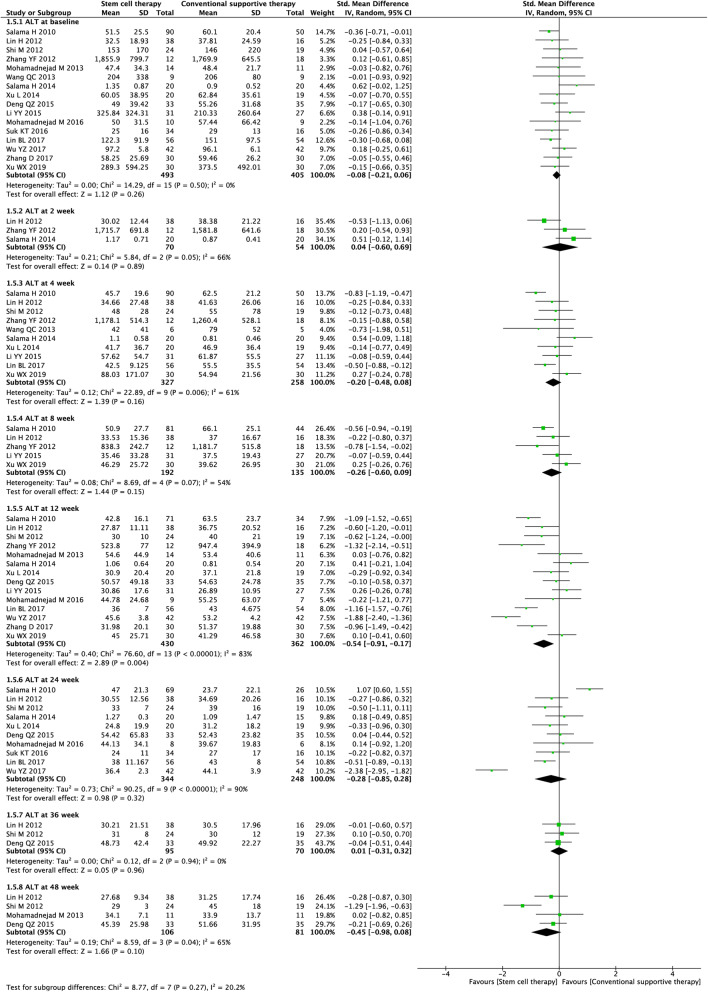
Forest plot of the comparison of the effect of stem cell therapy versus conventional treatment on alanine aminotransferase (ALT) level

Sixteen studies (3670 participants) were included in the analysis of the ALT level (Fig. 7). Before treatment, no significant difference was observed between the experimental and control groups (SMD = − 0.08, 95% CI − 0.21 to 0.06; *P* = 0.26). After treatment, stem cell therapy was associated with significantly lower ALT levels only at week 12 (SMD = − 0.54, 95% CI − 0.91 to − 0.17; *P* = 0.004).

We found substantial heterogeneity at most of the time points (*I*^*2*^ = 54–90%). By excluding the results of Lin et al. [23] at week 2, Salama et al. [21] at week 4, Xu et al. [43] at week 8, and Shi et al. [24] at week 48, sensitivity analyses showed lowered heterogeneity among the remaining studies (Additional file 2: Table S2). Publication bias was evaluated at weeks 4, 12, and 24, and the funnel plot and Egger’s test indicated no evident publication bias (Additional file 3 Fig. S1).

#### Coagulation function (PTA and INR)

**Fig. 8:**
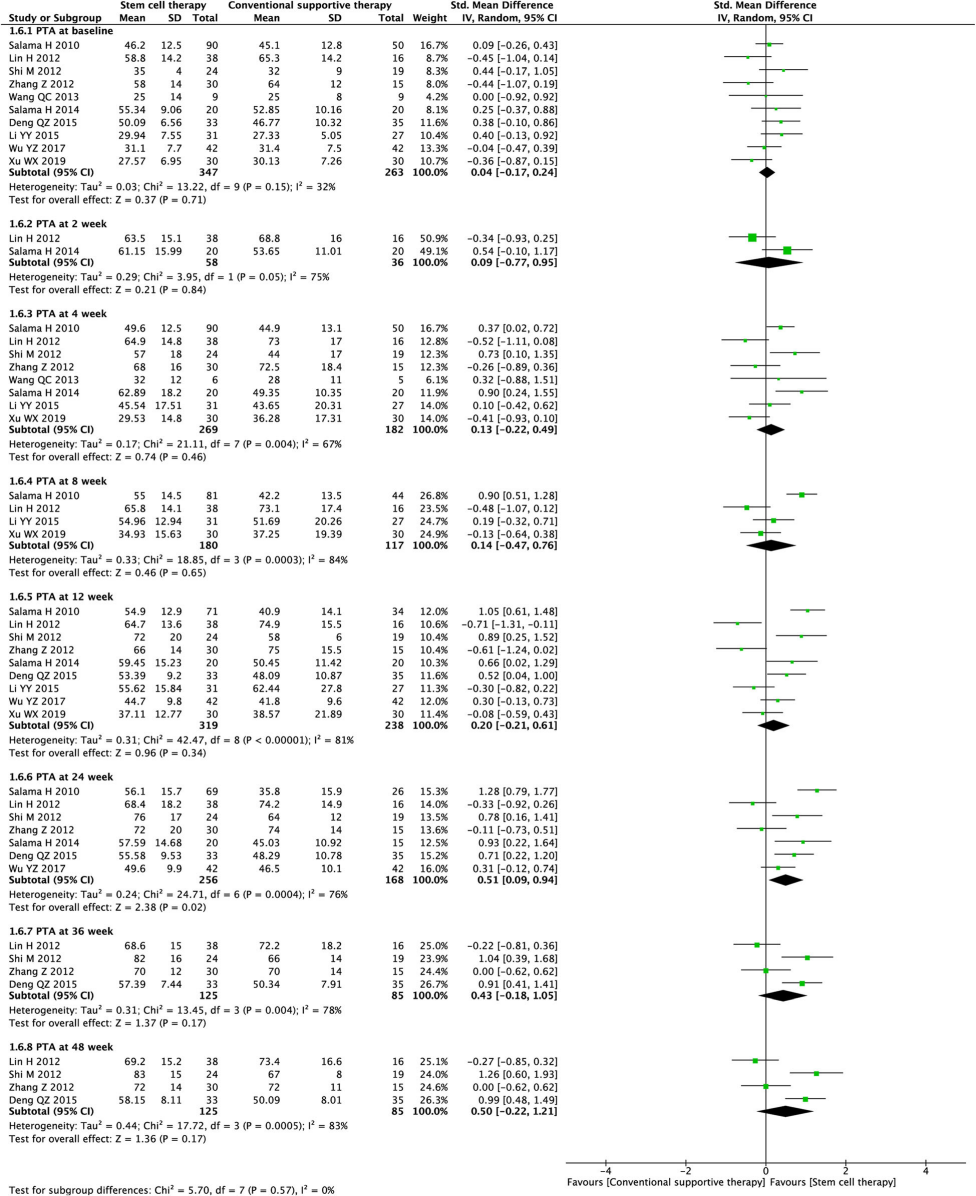
Forest plot of the comparison of the effect of stem cell therapy versus conventional treatment on prothrombin activity (PTA) level

**Fig. 9:**
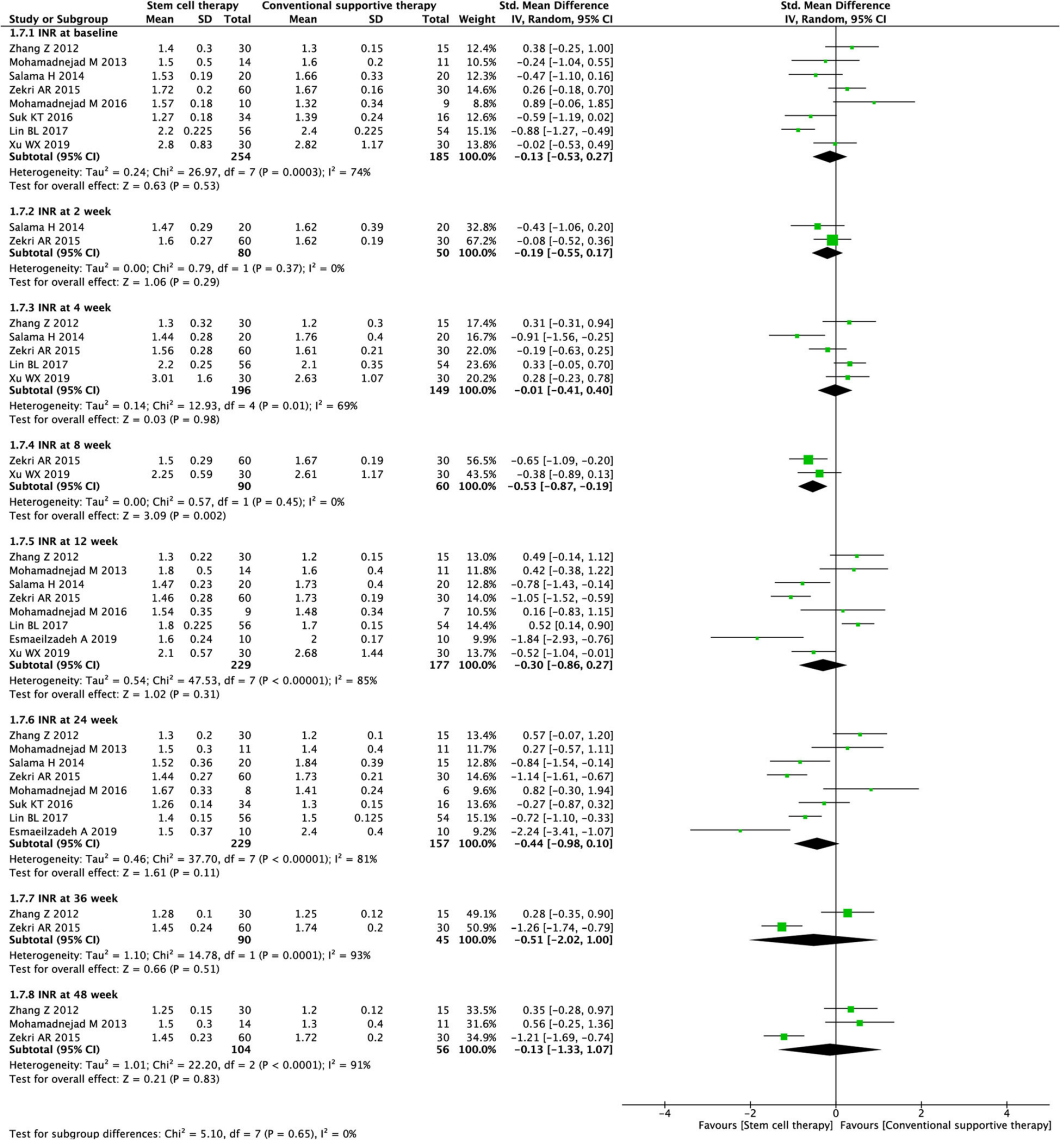
Forest plot of the comparison of the effect of stem cell therapy versus conventional treatment on international normalized ratio (INR) level

Ten studies with 2853 participants and 9 studies with 2151 participants were included in the analysis of the PTA level (Fig. 8) and INR level (Fig. 9), respectively. Before treatment, no significant difference in PTA level and INR level was observed between the experimental and control groups [(SMD = 0.04, 95% CI − 0.17 to 0.24; *P* = 0.71), (SMD = 0.13, 95% CI − 0.53 to 0.27; *P* = 0.53)]. After treatment, stem cell therapy was associated with significantly increased PTA level at week 24 (SMD = 0.51, 95% CI 0.09 to 0.94; *P* = 0.02) and lowered INR level at week 8 (SMD = − 0.53, 95% CI − 0.87 to − 0.19; *P* = 0.002).

We found substantial heterogeneity at most of the time points (*I*^*2*^ = 67–93%). By excluding the results of Salama et al. [21] at week 8 (PTA), Salama et al. [21] at week 4 (INR), and Zekri et al. [34] at week 48 (INR), sensitivity analyses showed lowered heterogeneity among the remaining studies (Additional file 2: Table S2). Due to the insufficient number of included studies, publication bias was not evaluated.

#### Subgroup analysis

**Fig. 10:**
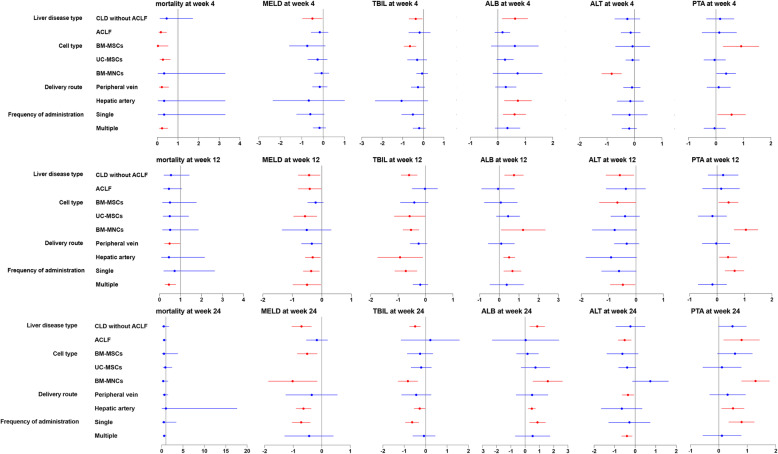
Subgroup analyses by the liver disease type, cell type, delivery route, and frequency of administration. Red indicates a significant improvement in the stem cell therapy group compared with the conventional treatment group; blue indicates no significant improvement. CLD chronic liver disease, ACLF acute-on-chronic liver failure, BM-MSC bone marrow-derived mesenchymal stem cell, UC-MSC umbilical cord-derived mesenchymal stem cell, BM-MNC bone marrow-derived mononuclear stem cell

We conducted subgroup analyses to explore whether the effects of stem cell therapy on mortality, MELD score, and TBIL, ALB, ALT, and PTA levels at weeks 4, 12, and 24 were influenced by different disease populations, cell type, delivery route, and administration frequency (Fig. 10).

#### Liver disease type (ACLF versus CLD without ACLF)

Compared with the conventional treatment group, stem cell therapy was associated with lower all-cause mortality in the ACLF subgroup, as indicated by decreased all-cause mortality at week 4. Stem cell therapy was associated with more improved liver functions in the CLD without ACLF subgroup, as indicated by decreased MELD scores, decreased TBIL levels, and increased ALB levels at weeks 4, 12, and 24. Stem cell therapy was associated with more improved liver functions in the ACLF subgroup, as indicated by decreased MELD score at week 12, decreased ALT level at week 24, and increased PTA level at week 24.

#### Cell type (BM-MSCs, UC-MSCs versus BM-MNCs)

Compared with the conventional treatment group, stem cell therapy was associated with lower all-cause mortality in the BM-MSC and US-MCS subgroups, as indicated by decreased all-cause mortality at week 4. Stem cell therapy was associated with more improved liver functions in the BM-MSC subgroup, as indicated by decreased MELD score at week 24, decreased TBIL level at week 4, decreased ALT levels at week 12, and increased PTA levels at weeks 4 and 12. Stem cell therapy was associated with more improved liver functions in the BM-MNC subgroup, as indicated by decreased MELD score at week 24, decreased TBIL level at weeks 12 and 24, increased ALB levels at weeks 12 and 24, decreased ALT levels at week 4, and increased PTA levels at weeks 12 and 24. Stem cell therapy was associated with more improved liver functions in the UC-MSC subgroup, as indicated by decreased MELD score at week 12 and TBIL level at week 12.

#### Delivery route (peripheral vein versus hepatic artery)

Compared with the conventional treatment group, stem cell therapy was associated with lower all-cause mortality in the peripheral vein administration subgroup, as indicated by decreased all-cause mortality at weeks 4 and 12. Stem cell therapy was associated with more improved liver functions in the hepatic artery administration subgroup, as indicated by decreased MELD scores and TBIL levels at weeks 12 and 24; increased ALB levels at weeks 4, 12, and 24; and increased PTA levels at weeks 12 and 24. Stem cell therapy was associated with more improved liver functions in the peripheral vein administration subgroup, as indicated by decreased ALT level at week 24.

#### Frequency of administration (single injection versus multiple injections)

Compared with the conventional treatment group, stem cell therapy was associated with lower all-cause mortality in the multiple injection subgroup, as indicated by decreased all-cause mortality at weeks 4 and 12. Stem cell therapy was associated with more improved liver functions in the single injection subgroup, as indicated by decreased MELD scores and TBIL levels at weeks 12 and 24; increased ALB levels at weeks 4, 12, and 24; and increased PTA levels at weeks 4, 12, and 24. Stem cell therapy was associated with more improved liver functions in the multiple injection subgroup, as indicated by decreased MELD score at week 12 and decreased ALT levels at weeks 12 and 24.

#### Adverse events associated with stem cell therapy

Five studies [27, 32, 35, 37, 39] reported that there were no procedural complications after cell infusion, while thirteen studies [20–22, 24–26, 28, 31, 33, 36, 38, 40, 43] reported adverse events of stem cell therapy, including fever, transient shivering, local pain, ecchymosis/hematoma, rash, diarrhea, chest tightness, and constipation, most of which resolved spontaneously (Additional file 5: Table S3).

## Discussion

In the present study, we produced a comprehensive meta-analysis of 24 randomized clinical trials to evaluate the therapeutic effects and safety of stem cell therapy in the treatment of patients with CLD. To our knowledge, it is the systematic review that includes the most RCTs up to now. Our study suggests compared with conventional treatment, stem cell therapy was associated with more favorable therapeutic effects, including lowered mortality and MELD scores, increased ALB levels, and decreased TBIL levels, while improvement in ALT, PTA, or INR was not evident. No serious adverse events related to the implantation of stem cells were reported. Overall, available evidence indicates that stem cell therapy is a safe and efficient treatment option for CLD.

Since safety is a major concern when initiating a new therapeutic strategy, our analysis evaluated the safety of stem cell therapy for treating CLD in terms of all-cause mortality and procedural adverse events. We find stem cell therapy significantly reduced all-cause mortality, with no serious adverse effect or death directly related to the implantation of stem cells themselves. Nevertheless, some potential risk of stem cell therapy must be cautiously considered, including immune reactivity, viral transmission, and tumorigenic potential [7, 44, 45]. Further high-quality clinical studies with larger sample size and longer follow-up period are still warranted to investigate the safety of stem cell therapy.

Liver disease population, cell type, delivery route, and injection frequency are highly variable among different studies, which will influence therapeutic effects of stem cell therapy [46]. Our subgroup analyses indicate that patients with ACLF had a short-term survival benefit from stem cell therapy, while other CLD patients had improved liver function. ACLF is a serious life-threatening disease and LT is the only effective treatment. Against this background, stem cell therapy can be a promising therapeutic option to temporarily support recipient through the limited survival time or waiting period until the spontaneous recovery of the native liver or availability of a suitable donor organ.

Of different cell types, MSCs especially show promise as an ideal cell resource for the treatment of liver disease [6, 44]. However, no clinical trial has directly compared different kinds of stem cells with regard to efficacy for CLD. Our study shows stem cells derived from the bone marrow (BM-MSCs and BM-MNCs) had superior therapeutic effects to UC-MSCs, which may be due to the different homing or migration ability of transplanted stem cells derived from different tissues [47].

Multiple infusions were considered to be associated with greater and sustained efficacy [34]. In contrast, one recent study demonstrated two-time injections of stem cells did not contribute to better therapeutic effects than a single injection [36]. This meta-analysis shows multiple injections only exerted greater beneficial effects on mortality and ALT levels, while a single administration could achieve more favorable effects, particularly on the MELD scores and TBIL, ALB, and TPA levels. Nonetheless, it is worth noting that the interval between the first and second infusions will have an important influence on the achievement of long-term therapeutic effects.

Across different trials, stem cells were delivered into the liver through peripheral intravenous, intrasplenic, hepatic arterial, or portal vein administrations. Our study shows hepatic arterial infusion was associated with better effects at more time points than intravenous infusion. It may be due to the different migration or homing ability of infused stem cells to the injured liver. Although peripheral intravenous infusion is an easy and convenient way with less-traumatic effects [30], systemic administration may cause the entrapment of a large number of cells within the capillaries, especially in the lungs [47]. In contrast, direct administration of cells into the liver through hepatic arterial infusion can significantly reduce the loss of cells in the circulation, thereby increasing the number of cells migrating to the injured sites [48, 49]. However, notably, hepatic arterial infusion is invasive and carries substantial risks including portal hypertensive bleeding and thrombosis following cell injection [50, 51].

Several limitations of the present meta-analysis are worth noting. Firstly, the majority of the included studies showed a high risk of bias. Secondly, the presence of substantial heterogeneity in some pooled estimate outcomes may hinder the establishment of robust conclusions and recommendations. Subgroup and sensitivity analyses did not provide a clear explanation, which suggested the heterogeneity may be due to true differences between studies. The degree of progression of liver disease (compensated or decompensated stage), liver disease types (viral hepatitis-related, autoimmune, alcoholic or other types of liver disease), and the purity, density, number, and quality of infused cells may be the key factors influencing the therapeutic efficacy of cell transplantation, which possibly contribute to some heterogeneity. However, available data did not allow us to assess whether these factors have an impact on outcomes. Thirdly, different trials evaluated therapeutic effects with different outcome parameters at different measurement time points, so it was difficult to summarize robust results using the limited statistical sample sizes at a specific time point.

Despite these limitations, our meta-analysis only included randomized clinical trials, while previous systematic reviews conducted pooled analyses of RCT and non-RCT. However, studies of different designs should not be analyzed in a combined manner; thus, our study theoretically could provide more reliable evidence than the previous ones, supporting stem cell therapy as a safe and effective treatment for CLD. Nonetheless, many factors still challenge the establishment of stem cell therapy as a definite treatment in patients with CLD [52]. The source, purity, density, and quality of stem cells and the dosage, route, and frequency of cell infusion are critical for therapeutic effects of stem cell therapy in the treatment of CLD. Hence, future preclinical and clinical researches should focus on the optimization of cell isolation, culture condition, and differentiation protocol; the determination of ideal cell source, cell dosage, injection frequency, and administration route; and the choice of therapeutic timing in various liver diseases. The prospects of stem cell therapy in the treatment of CLD will be determined by the outcomes of upcoming clinical studies.

## Conclusion

This meta-analysis suggests stem cell therapy is a safe and effective therapeutic approach for patients with CLD, while patients with ACLF benefit most in terms of improved short-term survival. A single injection administration with bone marrow-derived stem cell has superior therapeutic effects, and hepatic artery injection is the optimum cell delivery approach. There are significant heterogeneity and high risk of bias in existing studies; therefore, further high-quality randomized clinical studies are still in demand to acquire more solid evidence for the safety and efficacy of stem cell therapy in the treatment of CLD.

## Supplementary information

**Additional file 1: Table S1.** Search strategy

**Additional file 2: Table S2.** Results of sensitivity analyses with omission of one study at a time

**Additional file 3: Figure S1.** Funnel plots of mortality at weeks 4, 12, and 48; MELD at weeks 12 and 24; TBIL at weeks 4, 12 and 24; ALB at weeks 4, 12 and 24; and ALT at weeks 4, 12 and 24. Asymmetry was observed in the funnel plots of mortality at week 48 (*P* = 0.016) and TBIL at week 12 (*P* = 0.035).

**Additional file 4: Figure S2.** Symmetrical contour-enhanced funnel plots for mortality at week 48 and TBIL at week 12. For mortality at week 48, three hypothetical studies were filled: two plotted in the area of statistical significance and one in the area of statistical non-significance, indicating that the asymmetry in the funnel plot was partly caused by publication bias. For TBIL at week 12, no hypothetical studies were filled, indicating that the asymmetry in the funnel plot was not caused by publication bias.

**Additional file 5: Table S3.** Adverse events associated with stem cell therapy.

## Data Availability

Availability of data and materials can be assessed both in the “Methods” section, the “Results” section, and the “Additional files” section.

## Abbreviations

CLD: Chronic liver disease
ACLF: Acute-on-chronic liver failure
LT: Liver transplantation
RCT: Randomized controlled trial
CENTRAL: Cochrane Central Register of Controlled Trials
MELD: Model for end-stage liver disease
TBIL: Total bilirubin
ALB: Albumin
ALT: Alanine aminotransferase
PTA: Prothrombin activity
INR: International normalized ratio
SMD: Standardized mean difference
OR: Odds risk
CI: Confidence interval
BM-MSC: Bone marrow-derived mesenchymal stem cell
UC-MSC: Umbilical cord-derived mesenchymal stem cell
BM-MNC: Bone marrow-derived mononuclear stem cell
PBSC: Peripheral blood stem cell
