# Supplementary material for: Therapeutic effect and safety of stem cell therapy for chronic liver disease: a systematic review and meta-analysis of randomized controlled trials
Source: Stem Cell Res Ther. 2020 Sep 25;11:419. doi: 10.1186/s13287-020-01935-w (PMC7519526; doi:10.1186/s13287-020-01935-w)
Supplement: Supplementary file 1 — Additional file 1: Table S1. Search strategy [file 13287_2020_1935_MOESM1_ESM.docx]

**Additional file 1: Table S1.** Search strategy

| **MEDLINE (PubMed)** | 1946 to 16 March 2020 | #1 | (((liver or hepatic))) AND ((cirrhosis or fibrosis or disease or failure or cirrhotic)) |
| --- | --- | --- | --- |
|  |  | #2 | "Liver Diseases"[Mesh] |
|  |  | #3 | #1 or #2 |
|  |  | #4 | (stem cell* or multipotent cell* or mesenchymal stem cell* or multipotent stromal cell* or hematopoietic stem cell* or stem cell transplant*) |
|  |  | #5 | "Stem Cells"[Mesh] |
|  |  | #6 | "Stem Cell Transplantation"[Mesh] |
|  |  | #7 | #4 or #5 or #6 |
|  |  | #8 | #3 and #7 |
|  |  | #9 | (random* or blind* or placebo* or meta-analys*) |
|  |  | #10 | #8 and #9 |
|  |  | #11 | Filters: Clinical Trial; |
| **Ovid EMBASE** | 1974 to 16 March 2020 | 1 | ((liver or hepatic) and (cirrhosis or fibrosis or disease or failure or cirrhotic)).mp. [mp=title, abstract, heading word, drug trade name, original title, device manufacturer, drug manufacturer, device trade name, keyword, floating subheading word, candidate term word] |
|  |  | 2 | exp liver disease/ |
|  |  | 3 | 1 or 2 |
|  |  | 4 | (stem cell* or multipotent cell* or mesenchymal stem cell* or multipotent stromal cell* or hematopoietic stem cell* or stem cell transplant*).mp. [mp=title, abstract, heading word, drug trade name, original title, device manufacturer, drug manufacturer, device trade name, keyword, floating subheading word, candidate term word] |
|  |  | 5 | exp stem cells/ |
|  |  | 6 | exp stem cell transplantation/ |
|  |  | 7 | 4 or 5 or 6 |
|  |  | 8 | 3 and 7 |
|  |  | 9 | (random* or blind* or placebo* or meta-analys*).mp. [mp=title, abstract, heading word, drug trade name, original title, device manufacturer, drug manufacturer, device trade name, keyword, floating subheading word, candidate term word] |
|  |  | 10 | 8 and 9 |
|  |  | 11 | limit 10 to human |
| Cochrane Central Register of Controlled Trials (CENTRAL) in the Cochrane Library | 2020, Issue 3 | #1 | MeSH descriptor: [Stem Cells] explode all trees |
|  |  | #2 | MeSH descriptor: [Stem Cell Transplantation] explode all trees |
|  |  | #3 | “stem cell*” or “multipotent cell*” or “mesenchymal stem cell*” or “multipotent stromal cell*” or “hematopoietic stem cell*” or “stem cell transplant*” |
|  |  | #4 | #1 or #2 or #3 |
|  |  | #5 | MeSH descriptor: [Liver Diseases] explode all trees |
|  |  | #6 | (liver or hepatic) and (cirrhosis or fibrosis or disease or failure or cirrhotic) |
|  |  | #7 | #5 or #6 |
|  |  | #8 | #4 and #7 |
| ClinicalTrials.gov | 16 March 2020 |  | “Stem cells” and “liver” \|studies with Results |
