# Supplementary material for: Therapeutic effect and safety of stem cell therapy for chronic liver disease: a systematic review and meta-analysis of randomized controlled trials
Source: Stem Cell Res Ther. 2020 Sep 25;11:419. doi: 10.1186/s13287-020-01935-w (PMC7519526; doi:10.1186/s13287-020-01935-w)
Supplement: Supplementary file 2 — Additional file 2: Table S2. Results of sensitivity analyses with omission of one study at a time [file 13287_2020_1935_MOESM2_ESM.docx]

**Additional file 2: Table S2.** Results of sensitivity analyses with omission of one study at a time

|  | **Random-effects model (Mantel-Haenszel)** | | | |
| --- | --- | --- | --- | --- |
| **All-causes mortality at week 24** | **OR 95%-CI** | ***p*-value** | **tau^2^** | ***I^2^*** |
| Pooled estimate | 0.49 [0.21, 1.15] | 0.10 | 0.74 | 55% |
| Omitting Lyra AC 2010 | 0.47 [0.19, 1.17] | 0.11 | 0.81 | 59% |
| **Omitting Salama H 2010** | **0.64 [0.37, 1.11]** | **0.11** | **0.00** | **0%** |
| Omitting Mohamadnejad M 2013 | 0.41 [0.18, 0.92] | 0.03 | 0.57 | 51% |
| Omitting Salama H 2014 | 0.56 [0.23, 1.36] | 0.20 | 0.74 | 57% |
| Omitting Zekri AR 2014 | 0.47 [0.19, 1.17] | 0.10 | 0.81 | 59% |
| Omitting Mohamadnejad M 2016 | 0.48 [0.19, 1.19] | 0.11 | 0.82 | 60% |
| Omitting Lin BL 2017 | 0.54 [0.18, 1.66] | 0.28 | 1.29 | 60% |
| Omitting Newsome PN 2018 | 0.47 [0.19, 1.18] | 0.11 | 0.82 | 60% |
| Omitting Xu WX 2019 | 0.44 [0.17, 1.15] | 0.09 | 0.81 | 52% |
|  | **Random-effects model (Inverse-Variance)** | | | |
| **MELD at week 4** | **SMD 95%-CI** | ***p*-value** | **tau^2^** | ***I^2^*** |
| Pooled estimate | -0.33 [-0.67, 0.00] | 0.05 | 0.17 | 69% |
| Omitting Amer ME 2011 | -0.28 [-0.63, 0.07] | 0.12 | 0.17 | 69% |
| Omitting Shi M 2012 | -0.26 [-0.61, 0.08] | 0.13 | 0.15 | 67% |
| Omitting Wang QC 2013 | -0.35 [-0.70, 0.01] | 0.06 | 0.19 | 73% |
| Omitting Spahr L 2013 | -0.40 [-0.77, -0.04] | 0.03 | 0.19 | 70% |
| **Omitting Xu L 2014** | **-0.19 [-0.45, 0.08]** | **0.16** | **0.06** | **46%** |
| Omitting Li YY 2015 | -0.38 [-0.76, 0.01] | 0.05 | 0.21 | 72% |
| Omitting Zekri AR 2015 | -0.36 [-0.75, 0.04] | 0.08 | 0.23 | 73% |
| Omitting Lin BL 2017 | -0.40 [-0.78, -0.03] | 0.04 | 0.20 | 69% |
| Omitting Xu WX 2019 | -0.39 [-0.77, -0.01] | 0.04 | 0.20 | 71% |
|  | **Random-effects model (Inverse-Variance)** | | | |
| **MELD at week 12** | **SMD 95%-CI** | ***p*-value** | **tau^2^** | ***I^2^*** |
| Pooled estimate | -0.37 [-0.62, -0.12] | 0.003 | 0.10 | 57% |
| Omitting Shi M 2012 | -0.32 [-0.57, -0.08] | 0.01 | 0.08 | 53% |
| Omitting Mohamadnejad M 2013 | -0.40 [-0.66, -0.15] | 0.002 | 0.10 | 59% |
| Omitting Spahr L 2013 | -0.42 [-0.68, -0.16] | 0.002 | 0.09 | 56% |
| Omitting Xu L 2014 | -0.35 [-0.62, -0.08] | 0.01 | 0.11 | 60% |
| Omitting Deng QZ 2015 | -0.37 [-0.65, -0.09] | 0.01 | 0.12 | 61% |
| Omitting Li YY 2015 | -0.38 [-0.65, -0.10] | 0.008 | 0.12 | 61% |
| **Omitting Zekri AR 2015** | **-0.29 [-0.49, -0.08]** | **0.006** | **0.03** | **29%** |
| Omitting Mohamadnejad M 2016 | -0.41 [-0.66, -0.16] | 0.001 | 0.09 | 57% |
| Omitting Lin BL 2017 | -0.42 [-0.69, -0.16] | 0.002 | 0.10 | 54% |
| Omitting Wu YZ 2017 | -0.38 [-0.66, -0.10] | 0.008 | 0.12 | 61% |
| Omitting Xu WX 2019 | -0.37 [-0.65, -0.09] | 0.009 | 0.12 | 61% |
| **MELD at week 24** | **Random-effects model (Inverse-Variance)** | | | |
|  | **SMD 95%-CI** | ***p*-value** | **tau^2^** | ***I^2^*** |
| Pooled estimate | -0.57 [-0.92, -0.23] | 0.001 | 0.20 | 71% |
| Omitting Amer ME 2011 | -0.53 [-0.90, -0.16] | 0.005 | 0.22 | 73% |
| Omitting Mohamadnejad M 2013 | -0.67 [-1.00, -0.33] | < 0.001 | 0.16 | 67% |
| Omitting Xu L 2014 | -0.54 [-0.92, -0.16] | 0.005 | 0.23 | 73% |
| Omitting Deng QZ 2015 | -0.59 [-0.99, -0.20] | 0.003 | 0.25 | 73% |
| Omitting Zekri AR 2015 | -0.48 [-0.81, -0.15] | 0.005 | 0.15 | 62% |
| Omitting Mohamadnejad M 2016 | -0.64 [-0.98, -0.29] | < 0.001 | 0.19 | 71% |
| Omitting Suk KT 2016 | -0.60 [-0.98, -0.21] | 0.002 | 0.24 | 73% |
| Omitting Lin BL 2017 | -0.63 [-1.00, -0.26] | < 0.001 | 0.20 | 68% |
| Omitting Wu YZ 2017 | -0.53 [-0.91, -0.14] | 0.007 | 0.23 | 72% |
| Omitting Esmaeilzadeh A 2019 | -0.51 [-0.86, -0.16] | 0.004 | 0.19 | 71% |
| **MELD at week 48** | **Random-effects model (Inverse-Variance)** | | | |
|  | **SMD 95%-CI** | ***p*-value** | **tau^2^** | ***I^2^*** |
| Pooled estimate | -0.45 [-0.98, 0.08] | 0.10 | 0.13 | 62% |
| **Omitting Mohamadnejad M 2013** | **-0.70 [-1.03, -0.36]** | **< 0.001** | **0.00** | **0%** |
| Omitting Deng QZ 2015 | -0.22 [-1.23, 0.79] | 0.67 | 0.42 | 78% |
| Omitting Zekri AR 2015 | -0.23 [-1.29, 0.82] | 0.66 | 0.46 | 79% |
|  | **Random-effects model (Inverse-Variance)** | | | |
| **TBIL level at week 4** | **SMD 95%-CI** | ***p*-value** | **tau^2^** | ***I^2^*** |
| Pooled estimate | -0.31 [-0.58, -0.05] | 0.02 | 0.13 | 63% |
| Omitting Salama H 2010 | -0.35 [-0.65, -0.05] | 0.02 | 0.15 | 64% |
| Omitting Lin H 2012 | -0.35 [-0.63, -0.06] | 0.02 | 0.14 | 65% |
| Omitting Shi M 2012 | -0.30 [-0.59, -0.02] | 0.04 | 0.15 | 66% |
| **Omitting Zhang YF 2012** | **-0.22 [-0.44, 0.00]** | **0.05** | **0.06** | **47%** |
| Omitting Zhang Z 2012 | -0.32 [-0.61, -0.04] | 0.03 | 0.15 | 67% |
| Omitting Wang QC 2013 | -0.30 [-0.57, -0.02] | 0.03 | 0.14 | 66% |
| Omitting Salama H 2014 | -0.27 [-0.55, 0.00] | 0.05 | 0.13 | 64% |
| Omitting Xu L 2014 | -0.30 [-0.59, -0.02] | 0.04 | 0.14 | 66% |
| Omitting Li YY 2015 | -0.34 [-0.63, -0.05] | 0.02 | 0.15 | 66% |
| Omitting Zekri AR 2015 | -0.34 [-0.64, -0.04] | 0.02 | 0.15 | 66% |
| Omitting Lin BL 2017 | -0.27 [-0.54, 0.01] | 0.06 | 0.12 | 60% |
| Omitting Xu WX 2019 | -0.38 [-0.63, -0.13] | 0.003 | 0.09 | 54% |
|  | **Random-effects model (Inverse-Variance)** | | | |
| **TBIL level at week 8** | **SMD 95%-CI** | ***p*-value** | **tau^2^** | ***I^2^*** |
| Pooled estimate | -0.56 [-1.21, 0.08] | 0.09 | 0.53 | 88% |
| Omitting Salama H 2010 | -0.69 [-1.55, 0.16] | 0.11 | 0.81 | 90% |
| Omitting Lin H 2012 | -0.76 [-1.52, 0.00] | 0.05 | 0.62 | 90% |
| **Omitting Zhang YF 2012** | **-0.15 [-0.36, 0.05]** | **0.15** | **0.00** | **0%** |
| Omitting Li YY 2015 | -0.74 [-1.53, 0.06] | 0.07 | 0.69 | 90% |
| Omitting Zekri AR 2015 | -0.72 [-1.56, 0.11] | 0.09 | 0.76 | 91% |
| Omitting Xu WX 2019 | -0.75 [-1.54, 0.05] | 0.07 | 0.69 | 90% |
|  | **Random-effects model (Inverse-Variance)** | | | |
| **TBIL at week 12** | **SMD 95%-CI** | ***p*-value** | **tau^2^** | ***I^2^*** |
| Pooled estimate | -0.43 [-0.70, -0.17] | 0.001 | 0.21 | 73% |
| Omitting Salama H 2010 | -0.41 [-0.70, -0.13] | 0.004 | 0.22 | 73% |
| Omitting Lin H 2012 | -0.46 [-0.74, -0.18] | 0.001 | 0.22 | 74% |
| Omitting Shi M 2012 | -0.43 [-0.71, -0.14] | 0.003 | 0.22 | 74% |
| **Omitting Zhang YF 2012** | **-0.34 [-0.56, -0.12]** | **0.003** | **0.11** | **60%** |
| Omitting Zhang Z 2012 | -0.43 [-0.72, -0.15] | 0.003 | 0.22 | 74% |
| Omitting Mohamadnejad M 2013 | -0.43 [-0.71, -0.15] | 0.002 | 0.22 | 74% |
| Omitting Salama H 2014 | -0.41 [-0.68, -0.13] | 0.004 | 0.21 | 73% |
| Omitting Xu L 2014 | -0.44 [-0.72, -0.16] | 0.002 | 0.22 | 75% |
| Omitting Deng QZ 2015 | -0.47 [-0.75, -0.18] | 0.001 | 0.22 | 74% |
| Omitting Li YY 2015 | -0.46 [-0.74, -0.17] | 0.002 | 0.22 | 74% |
| Omitting Zekri AR 2015 | -0.45 [-0.74, -0.16] | 0.003 | 0.23 | 75% |
| Omitting Mohamadnejad M 2016 | -0.44 [-0.72, -0.16] | 0.002 | 0.21 | 75% |
| **Omitting Lin BL 2017** | **-0.50 [-0.73, -0.27]** | **< 0.001** | **0.11** | **58%** |
| Omitting Wu YZ 2017 | -0.40 [-0.68, -0.13] | 0.004 | 0.20 | 72% |
| Omitting Zhang D 2017 | -0.44 [-0.73, -0.16] | 0.002 | 0.23 | 75% |
| Omitting Xu WX 2019 | -0.47 [-0.75, -0.19] | 0.001 | 0.22 | 74% |
|  | **Random-effects model (Inverse-Variance)** | | | |
| **TBIL at week 24** | **SMD 95%-CI** | ***p*-value** | **tau^2^** | ***I^2^*** |
| Pooled estimate | -0.40 [-0.75, -0.05] | 0.02 | 0.34 | 80% |
| Omitting Salama H 2010 | -0.34 [-0.69, 0.01] | 0.06 | 0.31 | 78% |
| Omitting Lin H 2012 | -0.46 [-0.82, -0.09] | 0.02 | 0.35 | 81% |
| Omitting Shi M 2012 | -0.40 [-0.77, -0.02] | 0.04 | 0.36 | 81% |
| Omitting Zhang Z 2012 | -0.40 [-0.78, -0.03] | 0.03 | 0.37 | 81% |
| Omitting Mohamadnejad M 2013 | -0.39 [-0.75, -0.02] | 0.04 | 0.35 | 81% |
| Omitting Salama H 2014 | -0.35 [-0.70, 0.01] | 0.05 | 0.32 | 80% |
| Omitting Xu L 2014 | -0.40 [-0.79, -0.03] | 0.03 | 0.36 | 81% |
| Omitting Deng QZ 2015 | -0.43 [-0.82, -0.05] | 0.03 | 0.38 | 81% |
| Omitting Zekri AR 2015 | -0.40 [-0.79, -0.02] | 0.04 | 0.38 | 81% |
| Omitting Mohamadnejad M 2016 | -0.39 [-0.76, -0.03] | 0.03 | 0.35 | 81% |
| Omitting Suk KT 2016 | -0.44 [-0.81, -0.06] | 0.02 | 0.36 | 81% |
| **Omitting Lin BL 2017** | **-0.49 [-0.74, -0.24]** | **< 0.01** | **0.11** | **54%** |
| Omitting Wu YZ 2017 | -0.40 [-0.78, -0.01] | 0.04 | 0.38 | 81% |
| Omitting Esmaeilzadeh A 2019 | -0.32 [-0.66, 0.02] | 0.06 | 0.29 | 79% |
|  | **Random-effects model (Inverse-Variance)** | | | |
| **TBIL at week 36** | **SMD 95%-CI** | ***p*-value** | **tau^2^** | ***I^2^*** |
| Pooled estimate | -0.31 [-0.71, 0.08] | 0.12 | 0.12 | 62% |
| Omitting Lin H 2012 | -0.42 [-0.86, 0.03] | 0.07 | 0.13 | 63% |
| **Omitting Shi M 2012** | **-0.15 [-0.40, 0.11]** | **0.27** | **0.00** | **0%** |
| Omitting Zhang Z 2012 | -0.34 [-0.83, 0.16] | 0.18 | 0.18 | 71% |
| Omitting Deng QZ 2015 | -0.40 [-0.89, 0.09] | 0.11 | 0.17 | 66% |
| Omitting Zekri AR 2015 | -0.31 [-0.84, 0.23] | 0.26 | 0.21 | 71% |
|  | **Random-effects model (Inverse-Variance)** | | | |
| **ALB level at week 2** | **SMD 95%-CI** | ***p*-value** | **tau^2^** | ***I^2^*** |
| Pooled estimate | 0.69 [0.03, 1.35] | 0.04 | 0.24 | 71% |
| Omitting Zhang YF 2012 | 0.58 [-0.33, 1.48] | 0.21 | 0.34 | 81% |
| Omitting Salama H 2014 | 0.51 [-0.31, 1.33] | 0.22 | 0.25 | 71% |
| **Omitting Zekri AR 2015** | **1.04 [0.53, 1.55]** | **< 0.001** | **0.00** | **0%** |
|  | **Random-effects model (Inverse-Variance)** | | | |
| **ALB level at week 4** | **SMD 95%-CI** | ***p*-value** | **tau^2^** | ***I^2^*** |
| Pooled estimate | 0.40 [0.13, 0.66] | 0.003 | 0.11 | 59% |
| Omitting Salama H 2010 | 0.42 [0.12, 0.73] | 0.007 | 0.14 | 63% |
| Omitting Shi M 2012 | 0.39 [0.10, 0.68] | 0.007 | 0.12 | 63% |
| Omitting Zhang YF 2012 | 0.37 [0.09, 0.64] | 0.009 | 0.11 | 61% |
| Omitting Zhang Z 2012 | 0.45 [0.19, 0.72] | < 0.001 | 0.09 | 56% |
| Omitting Wang QC 2013 | 0.40 [0.13, 0.68] | 0.004 | 0.12 | 63% |
| **Omitting Salama H 2014** | **0.31 [0.08, 0.54]** | **0.008** | **0.06** | **44%** |
| Omitting Xu L 2014 | 0.37 [0.09, 0.65] | 0.009 | 0.12 | 62% |
| Omitting Li YY 2015 | 0.44 [0.16, 0.72] | 0.002 | 0.12 | 61% |
| Omitting Zekri AR 2015 | 0.34 [0.07, 0.61] | 0.01 | 0.10 | 55% |
| Omitting Lin BL 2017 | 0.46 [0.19, 0.73] | < 0.001 | 0.09 | 53% |
| Omitting Xu WX 2019 | 0.39 [0.10, 0.69] | 0.008 | 0.13 | 63% |
| **at week 8** | **Random-effects model (Inverse-Variance)** | | | |
| **ALB level at week 8** | **SMD 95%-CI** | ***p*-value** | **tau^2^** | ***I^2^*** |
| Pooled estimate | 0.61 [0.11, 1.12] | 0.02 | 0.26 | 80% |
| Omitting Salama H 2010 | 0.55 [-0.13, 1.24] | 0.11 | 0.41 | 84% |
| Omitting Zhang YF 2012 | 0.58 [-0.02, 1.18] | 0.06 | 0.32 | 85% |
| Omitting Li YY 2015 | 0.73 [0.14, 1.32] | 0.01 | 0.28 | 81% |
| Omitting Zekri AR 2015 | 0.42 [-0.05, 0.89] | 0.08 | 0.16 | 69% |
| Omitting Xu WX 2019 | 0.79 [0.30, 1.28] | 0.001 | 0.17 | 72% |
|  | **Random-effects model (Inverse-Variance)** | | | |
| **ALB level at week 12** | **SMD 95%-CI** | ***p*-value** | **tau^2^** | ***I^2^*** |
| Pooled estimate | 0.40 [-0.05, 0.86] | 0.08 | 0.65 | 89% |
| Omitting Salama H 2010 | 0.32 [-0.13, 0.77] | 0.16 | 0.58 | 87% |
| Omitting Shi M 2012 | 0.34 [-0.13, 0.81] | 0.15 | 0.65 | 89% |
| Omitting Zhang YF 2012 | 0.36 [-0.12, 0.83] | 0.14 | 0.66 | 90% |
| Omitting Zhang Z 2012 | 0.40 [-0.09, 0.88] | 0.11 | 0.70 | 90% |
| Omitting Mohamadnejad M 2013 | 0.49 [0.02, 0.95] | 0.04 | 0.63 | 89% |
| Omitting Salama H 2014 | 0.34 [-0.13, 0.81] | 0.16 | 0.65 | 89% |
| Omitting Xu L 2014 | 0.39 [-0.09, 0.87] | 0.11 | 0.69 | 90% |
| Omitting Deng QZ 2015 | 0.40 [-0.09, 0.90] | 0.11 | 0.73 | 90% |
| Omitting Li YY 2015 | 0.45 [-0.03, 0.93] | 0.07 | 0.68 | 89% |
| Omitting Zekri AR 2015 | 0.32 [-0.13, 0.78] | 0.16 | 0.59 | 88% |
| Omitting Mohamadnejad M 2016 | 0.46 [-0.01, 0.93] | 0.05 | 0.65 | 90% |
| Omitting Lin BL 2017 | 0.53 [0.14, 0.91] | 0.007 | 0.40 | 82% |
| Omitting Wu YZ 2017 | 0.41 [-0.09, 0.91] | 0.11 | 0.75 | 90% |
| Omitting Xu WX 2019 | 0.45 [-0.03, 0.93] | 0.07 | 0.69 | 89% |
|  | **Random-effects model (Inverse-Variance)** | | | |
| **ALB level at week 24** | **SMD 95%-CI** | ***p*-value** | **tau^2^** | ***I^2^*** |
| Pooled estimate | 0.62 [0.03, 1.21] | 0.04 | 1.05 | 92% |
| Omitting Salama H 2010 | 0.50 [-0.07, 1.08] | 0.09 | 0.91 | 91% |
| Omitting Shi M 2012 | 0.57 [-0.05, 1.20] | 0.07 | 1.10 | 92% |
| Omitting Zhang Z 2012 | 0.66 [0.02, 1.29] | 0.14 | 1.14 | 93% |
| Omitting Mohamadnejad M 2013 | 0.75 [0.15, 1.35] | 0.01 | 1.01 | 92% |
| Omitting Salama H 2014 | 0.53 [-0.07, 1.14] | 0.09 | 1.04 | 92% |
| Omitting Xu L 2014 | 0.61 [-0.02, 1.25] | 0.06 | 1.13 | 93% |
| Omitting Deng QZ 2015 | 0.62 [-0.03, 1.27] | 0.06 | 1.20 | 93% |
| Omitting Zekri AR 2015 | 0.53 [-0.07, 1.14] | 0.08 | 1.01 | 91% |
| Omitting Mohamadnejad M 2016 | 0.70 [0.09, 1.31] | 0.03 | 1.06 | 92% |
| Omitting Suk KT 2016 | 0.65 [0.01, 1.29] | 0.05 | 1.15 | 93% |
| Omitting Lin BL 2017 | 0.78 [0.31, 1.26] | 0.001 | 0.58 | 85% |
| Omitting Wu YZ 2017 | 0.65 [-0.01, 1.31] | 0.05 | 1.23 | 93% |
| Omitting Esmaeilzadeh A 2019 | 0.53 [-0.08, 1.13] | 0.09 | 1.03 | 92% |
|  | **Random-effects model (Inverse-Variance)** | | | |
| **ALB level at week 36** | **SMD 95%-CI** | ***p*-value** | **tau^2^** | ***I^2^*** |
| Pooled estimate | 1.42 [0.56, 2.28] | 0.001 | 0.66 | 88% |
| Omitting Shi M 2012 | 0.99 [0.35, 1.64] | 0.002 | 0.24 | 76% |
| Omitting Zhang Z 2012 | 1.70 [0.60, 2.79] | 0.002 | 0.83 | 90% |
| Omitting Deng QZ 2015 | 1.69 [0.56, 2.82] | 0.003 | 0.87 | 89% |
| Omitting Zekri AR 2015 | 1.38 [0.15, 2.60] | 0.03 | 1.06 | 91% |
|  | **Random-effects model (Inverse-Variance)** | | | |
| **ALB level at week 48** | **SMD 95%-CI** | ***p*-value** | **tau^2^** | ***I^2^*** |
| Pooled estimate | 0.95 [0.07, 1.83] | 0.03 | 0.88 | 89% |
| Omitting Shi M 2012 | 0.64 [-0.31, 1.58] | 0.19 | 0.81 | 89% |
| Omitting Zhang Z 2012 | 0.90 [-0.24, 2.03] | 0.12 | 1.21 | 92% |
| **Omitting Mohamadnejad M 2013** | **1.41 [0.86, 1.96]** | **< 0.001** | **0.22** | **71%** |
| Omitting Deng QZ 2015 | 0.95 [-0.26, 2.16] | 0.12 | 1.38 | 92% |
| Omitting Zekri AR 2015 | 0.77 [-0.38, 1.92] | 0.19 | 1.24 | 91% |
|  | **Random-effects model (Inverse-Variance)** | | | |
| **ALT level at week 2** | **SMD 95%-CI** | ***p*-value** | **tau^2^** | ***I^2^*** |
| Pooled estimate | 0.04 [-0.60, 0.69] | 0.89 | 0.21 | 66% |
| **Omitting Lin H 2012** | **0.38 [-0.10, 0.85]** | **0.12** | **0.00** | **0%** |
| Omitting Zhang YF 2012 | -0.02 [-1.04, 1.00] | 0.97 | 0.44 | 82% |
| Omitting Salama H 2014 | -0.20 [-0.91, 0.51] | 0.58 | 0.15 | 56% |
|  | **Random-effects model (Inverse-Variance)** | | | |
| **ALT level at week 4** | **SMD 95%-CI** | ***p*-value** | **tau^2^** | ***I^2^*** |
| Pooled estimate | -0.20 [-0.48, 0.08] | 0.16 | 0.12 | 61% |
| **Omitting Salama H 2010** | **-0.11 [-0.34, 0.13]** | **0.37** | **0.04** | **30%** |
| Omitting Lin H 2012 | -0.19 [-0.51, 0.12] | 0.23 | 0.14 | 65% |
| Omitting Shi M 2012 | -0.21 [-0.52, 0.11] | 0.20 | 0.14 | 65% |
| Omitting Zhang YF 2012 | -0.20 [-0.51, 0.10] | 0.20 | 0.13 | 65% |
| Omitting Wang QC 2013 | -0.18 [-0.47, 0.12] | 0.24 | 0.12 | 64% |
| Omitting Salama H 2014 | -0.28 [-0.54, -0.03] | 0.03 | 0.07 | 50% |
| Omitting Xu L 2014 | -0.20 [-0.51, 0.11] | 0.20 | 0.14 | 65% |
| Omitting Li YY 2015 | -0.21 [-0.53, 0.10] | 0.18 | 0.14 | 64% |
| Omitting Lin BL 2017 | -0.15 [-0.47, 0.17] | 0.35 | 0.14 | 62% |
| Omitting Xu WX 2019 | -0.26 [-0.55, 0.02] | 0.07 | 0.10 | 55% |
|  | **Random-effects model (Inverse-Variance)** | | | |
| **ALT level at week 8** | **SMD 95%-CI** | ***p*-value** | **tau^2^** | ***I^2^*** |
| Pooled estimate | -0.26 [-0.60, 0.09] | 0.15 | 0.08 | 54% |
| Omitting Salama H 2010 | -0.14 [-0.51, 0.24] | 0.47 | 0.06 | 40% |
| Omitting Lin H 2012 | -0.27 [-0.71, 0.17] | 0.23 | 0.13 | 65% |
| Omitting Zhang YF 2012 | -0.18 [-0.54 0.19] | 0.35 | 0.08 | 56% |
| Omitting Li YY 2015 | -0.31 [-0.74, 0.13] | 0.17 | 0.12 | 62% |
| **Omitting Xu WX 2019** | **-0.40 [-0.68, -0.11]** | **0.006** | **0.01** | **17%** |
|  | **Random-effects model (Inverse-Variance)** | | | |
| **ALT level at week 12** | **SMD 95%-CI** | ***p*-value** | **tau^2^** | ***I^2^*** |
| Pooled estimate | -0.54 [-0.91, -0.17] | 0.004 | 0.40 | 83% |
| Omitting Salama H 2010 | -0.50 [-0.89, -0.10] | 0.01 | 0.42 | 83% |
| Omitting Lin H 2012 | -0.54 [-0.93, -0.14] | 0.008 | 0.44 | 84% |
| Omitting Shi M 2012 | -0.54 [-0.93, -0.14] | 0.008 | 0.43 | 84% |
| Omitting Zhang YF 2012 | -0.49 [-0.87, -0.11] | 0.01 | 0.40 | 84% |
| Omitting Mohamadnejad M 2013 | -0.58 [-0.97, -0.20] | 0.003 | 0.41 | 84% |
| Omitting Salama H 2014 | -0.62 [-0.98, -0.25] | 0.001 | 0.36 | 82% |
| Omitting Xu L 2014 | -0.56 [-0.96, -0.17] | 0.005 | 0.43 | 84% |
| Omitting Deng QZ 2014 | -0.58 [-0.97, -0.19] | 0.004 | 0.42 | 83% |
| Omitting Li YY 2015 | -0.61 [-0.98, -0.24] | 0.001 | 0.36 | 81% |
| Omitting Mohamadnejad M 2016 | -0.56 [-0.95, -0.18] | 0.004 | 0.41 | 84% |
| Omitting Lin BL 2017 | -0.49 [-0.88, -0.10] | 0.01 | 0.41 | 82% |
| Omitting Wu YZ 2017 | -0.44 [-0.77, -0.11] | 0.009 | 0.27 | 77% |
| Omitting Zhang D 2017 | -0.51 [-0.91, -0.11] | 0.01 | 0.43 | 84% |
| Omitting Xu WX 2019 | -0.60 [-0.98, -0.22] | 0.002 | 0.39 | 82% |
|  | **Random-effects model (Inverse-Variance)** | | | |
| **ALT level at week 24** | **SMD 95%-CI** | ***p*-value** | **tau^2^** | ***I^2^*** |
| Pooled estimate | -0.28 [-0.85, 0.28] | 0.32 | 0.73 | 90% |
| Omitting Salama H 2010 | -0.45 [-0.95, 0.06] | 0.08 | 0.50 | 85% |
| Omitting Lin H 2012 | -0.29 [-0.91, 0.34] | 0.37 | 0.83 | 91% |
| Omitting Shi M 2012 | -0.26 [-0.88, 0.37] | 0.42 | 0.82 | 91% |
| Omitting Salama H 2014 | -0.33 [-0.95, 0.28] | 0.29 | 0.79 | 91% |
| Omitting Xu L 2014 | -0.28 [-0.90, 0.35] | 0.38 | 0.82 | 91% |
| Omitting Deng QZ 2014 | -0.32 [-0.96, 0.32] | 0.32 | 0.85 | 91% |
| Omitting Mohamadnejad M 2016 | -0.32 [-0.92, 0.28] | 0.29 | 0.76 | 91% |
| Omitting Suk KT 2016 | -0.29 [-0.92, 0.34] | 0.37 | 0.83 | 91% |
| Omitting Lin BL 2017 | -0.26 [-0.92, 0.41] | 0.45 | 0.92 | 91% |
| Omitting Wu YZ 2019 | -0.05 [-0.42, 0.33] | 0.81 | 0.24 | 74% |
|  | **Random-effects model (Inverse-Variance)** | | | |
| **ALT level at week 48** | **SMD 95%-CI** | ***p*-value** | **tau^2^** | ***I^2^*** |
| Pooled estimate | -0.45 [-0.98, 0.08] | 0.10 | 0.19 | 65% |
| Omitting Lin H 2012 | -0.50 [-1.27, 0.26] | 0.20 | 0.34 | 76% |
| **Omitting Shi M 2012** | **-0.20 [-0.54, 0.14]** | **0.25** | **0.00** | **0%** |
| Omitting Mohamadnejad M 2013 | -0.57 [-1.20, 0.07] | 0.08 | 0.23 | 73% |
| Omitting Deng QZ 2015 | -0.54 [-1.30, 0.22] | 0.17 | 0.33 | 73% |
|  | **Random-effects model (Inverse-Variance)** | | | |
| **PTA level at week 4** | **SMD 95%-CI** | ***p*-value** | **tau^2^** | ***I^2^*** |
| Pooled estimate | 0.13 [-0.22, 0.49] | 0.46 | 0.17 | 67% |
| Omitting Salama H 2010 | 0.09 [-0.33, 0.51] | 0.67 | 0.21 | 68% |
| Omitting Lin H 2012 | 0.23 [-0.13, 0.58] | 0.21 | 0.13 | 62% |
| Omitting Shi M 2012 | 0.05 [-0.32, 0.42] | 0.79 | 0.15 | 66% |
| Omitting Zhang Z 2012 | 0.19 [-0.20, 0.58] | 0.34 | 0.18 | 69% |
| Omitting Wang QC 2013 | 0.12 [-0.26, 0.50] | 0.53 | 0.18 | 71% |
| Omitting Salama H 2014 | 0.03 [-0.31, 0.38] | 0.86 | 0.13 | 61% |
| Omitting Li YY 2015 | 0.14 [-0.28, 0.56] | 0.51 | 0.21 | 72% |
| Omitting Xu WX 2019 | 0.22 [-0.14, 0.59] | 0.23 | 0.14 | 62% |
|  | **Random-effects model (Inverse-Variance)** | | | |
| **PTA level at week 8** | **SMD 95%-CI** | ***p*-value** | **tau^2^** | ***I^2^*** |
| Pooled estimate | 0.14 [-0.47, 0.76] | 0.65 | 0.33 | 84% |
| **Omitting Salama H 2010** | **-0.12 [-0.48, 0.25]** | **0.53** | **0.03** | **28%** |
| Omitting Lin H 2012 | 0.34 [-0.30, 0.98] | 0.30 | 0.26 | 82% |
| Omitting Li YY 2015 | 0.12 [-0.74, 0.98] | 0.79 | 0.52 | 89% |
| Omitting Xu WX 2019 | 0.23 [-0.56, 1.02] | 0.57 | 0.42 | 87% |
|  | **Random-effects model (Inverse-Variance)** | | | |
| **PTA level at week 12** | **SMD 95%-CI** | ***p*-value** | **tau^2^** | ***I^2^*** |
| Pooled estimate | 0.20 [-0.21, 0.61] | 0.34 | 0.31 | 81% |
| Omitting Salama H 2010 | 0.08 [-0.30, 0.46] | 0.67 | 0.22 | 74% |
| Omitting Lin H 2012 | 0.31 [-0.09, 0.70] | 0.13 | 0.25 | 78% |
| Omitting Shi M 2012 | 0.12 [-0.31, 0.55] | 0.59 | 0.31 | 82% |
| Omitting Zhang Z 2012 | 0.29 [-0.12, 0.70] | 0.16 | 0.28 | 80% |
| Omitting Salama H 2014 | 0.14 [-0.30, 0.59] | 0.52 | 0.34 | 83% |
| Omitting Deng QZ 2015 | 0.16 [-0.30, 0.62] | 0.51 | 0.36 | 83% |
| Omitting Li YY 2015 | 0.26 [-0.17, 0.70] | 0.24 | 0.32 | 81% |
| Omitting Wu YZ 2017 | 0.18 [-0.29, 0.66] | 0.45 | 0.39 | 83% |
| Omitting Xu WX 2019 | 0.23 [-0.22, 0.69] | 0.31 | 0.35 | 83% |
|  | **Random-effects model (Inverse-Variance)** | | | |
| **PTA level at week 24** | **SMD 95%-CI** | ***p*-value** | **tau^2^** | ***I^2^*** |
| Pooled estimate | 0.51 [0.09, 0.94] | 0.02 | 0.24 | 76% |
| Omitting Salama H 2010 | 0.37 [-0.01, 0.75] | 0.06 | 0.14 | 63% |
| Omitting Lin H 2012 | 0.65 [0.26, 1.04] | 0.001 | 0.16 | 67% |
| Omitting Shi M 2012 | 0.47 [-0.01, 0.95] | 0.06 | 0.29 | 79% |
| Omitting Zhang Z 2012 | 0.61 [0.17, 1.05] | 0.007 | 0.23 | 75% |
| Omitting Salama H 2014 | 0.45 [-0.02, 0.92] | 0.06 | 0.27 | 79% |
| Omitting Deng QZ 2015 | 0.48 [-0.03, 0.98] | 0.06 | 0.31 | 79% |
| Omitting Wu YZ 2017 | 0.55 [0.04, 1.06] | 0.03 | 0.32 | 79% |
|  | **Random-effects model (Inverse-Variance)** | | | |
| **PTA level at week 36** | **SMD 95%-CI** | ***p*-value** | **tau^2^** | ***I^2^*** |
| Pooled estimate | 0.43 [-0.18, 1.05] | 0.17 | 0.31 | 78% |
| Omitting Lin H 2012 | 0.66 [0.04, 1.27] | 0.04 | 0.20 | 70% |
| Omitting Shi M 2012 | 0.24 [-0.47, 0.96] | 0.50 | 0.32 | 79% |
| Omitting Zhang Z 2012 | 0.57 [-0.20, 1.34] | 0.14 | 0.38 | 81% |
| Omitting Deng QZ 2015 | 0.26 [-0.48, 1.01] | 0.49 | 0.34 | 77% |
|  | **Random-effects model (Inverse-Variance)** | | | |
| **PTA level at week 48** | **SMD 95%-CI** | ***p*-value** | **tau^2^** | ***I^2^*** |
| Pooled estimate | 0.50 [-0.22, 1.21] | 0.17 | 0.44 | 83% |
| Omitting Lin H 2012 | 0.75 [0.03, 1.47] | 0.04 | 0.31 | 77% |
| Omitting Shi M 2012 | 0.26 [-0.54, 1.05] | 0.53 | 0.40 | 83% |
| Omitting Zhang Z 2012 | 0.66 [-0.24, 1.56] | 0.15 | 0.54 | 86% |
| Omitting Deng QZ 2015 | 0.32 [-0.58, 1.22] | 0.48 | 0.53 | 84% |
|  | **Random-effects model (Inverse-Variance)** | | | |
| **INR level at week 4** | **SMD 95%-CI** | ***p*-value** | **tau^2^** | ***I^2^*** |
| Pooled estimate | -0.01 [-0.41, 0.40] | 0.98 | 0.14 | 69% |
| Omitting Zhang Z 2012 | -0.08 [-0.57, 0.40] | 0.74 | 0.18 | 75% |
| **Omitting Salama H 2014** | **0.17 [-0.09, 0.43]** | **0.20** | **0.01** | **18%** |
| Omitting Zekri AR 2015 | 0.04 [-0.48, 0.55] | 0.89 | 0.20 | 74% |
| Omitting Lin BL 2017 | -0.11 [-0.61, 0.38] | 0.66 | 0.18 | 69% |
| Omitting Xu WX 2019 | -0.08 [-0.59, 0.42] | 0.74 | 0.20 | 75% |
|  | **Random-effects model (Inverse-Variance)** | | | |
| **INR level at week 12** | **SMD 95%-CI** | ***p*-value** | **tau^2^** | ***I^2^*** |
| Pooled estimate | -0.30 [-0.86, 0.27] | 0.31 | 0.54 | 85% |
| Omitting Zhang Z 2012 | -0.41 [-1.03, 0.21] | 0.19 | 0.57 | 86% |
| Omitting Mohamadnejad M 2013 | -0.39 [-1.01, 0.23] | 0.21 | 0.57 | 87% |
| Omitting Salama H 2014 | -0.23 [-0.85, 0.40] | 0.48 | 0.59 | 86% |
| Omitting Zekri AR 2015 | -0.16 [-0.73, 0.40] | 0.57 | 0.44 | 81% |
| Omitting Mohamadnejad M 2016 | -0.35 [-0.97, 0.27] | 0.26 | 0.58 | 87% |
| Omitting Lin BL 2017 | -0.43 [-0.99, 0.13] | 0.13 | 0.43 | 79% |
| Omitting Esmaeilzadeh A 2019 | -0.13 [-0.68, 0.43] | 0.65 | 0.46 | 84% |
| Omitting Xu WX 2019 | -0.26 [-0.93, 0.40] | 0.43 | 0.66 | 87% |
|  | **Random-effects model (Inverse-Variance)** | | | |
| **INR level at week 24** | **SMD 95%-CI** | ***p*-value** | **tau^2^** | ***I^2^*** |
| Pooled estimate | -0.44 [-0.98, 0.10] | 0.11 | 0.46 | 81% |
| Omitting Zhang Z 2012 | -0.60 [-1.11, -0.09] | 0.02 | 0.32 | 75% |
| Omitting Mohamadnejad M 2013 | -0.53 [-1.11, 0.04] | 0.07 | 0.46 | 82% |
| Omitting Salama H 2014 | -0.38 [-0.99, 0.23] | 0.22 | 0.53 | 84% |
| Omitting Zekri AR 2015 | -0.32 [-0.91, 0.27] | 0.29 | 0.47 | 80% |
| Omitting Mohamadnejad M 2016 | -0.57 [-1.11, -0.03] | 0.04 | 0.41 | 81% |
| Omitting Suk KT 2016 | -0.46 [-1.09, 0.16] | 0.14 | 0.55 | 84% |
| Omitting Lin BL 2017 | -0.39 [-1.07, 0.28] | 0.25 | 0.66 | 84% |
| Omitting Esmaeilzadeh A 2019 | -0.27 [-0.78, 0.25] | 0.31 | 0.37 | 80% |
|  | **Random-effects model (Inverse-Variance)** | | | |
| **INR level at week 48** | **SMD 95%-CI** | ***p*-value** | **tau^2^** | ***I^2^*** |
| Pooled estimate | -0.13 [-1.33, 1.07] | 0.83 | 1.01 | 91% |
| Omitting Zhang Z 2012 | -0.36 [-2.09, 1.37] | 0.68 | 1.45 | 93% |
| Omitting Mohamadnejad M 2013 | -0.45 [-1.98, 1.08] | 0.57 | 1.14 | 93% |
| **Omitting Zekri AR 2015** | **0.43 [-0.07, 0.92]** | **0.09** | **0.00** | **0%** |
