# Supplementary figures and images for: Therapeutic effect and safety of stem cell therapy for chronic liver disease: a systematic review and meta-analysis of randomized controlled trials
Source: Stem Cell Res Ther. 2020 Sep 25;11:419. doi: 10.1186/s13287-020-01935-w (PMC7519526; doi:10.1186/s13287-020-01935-w)

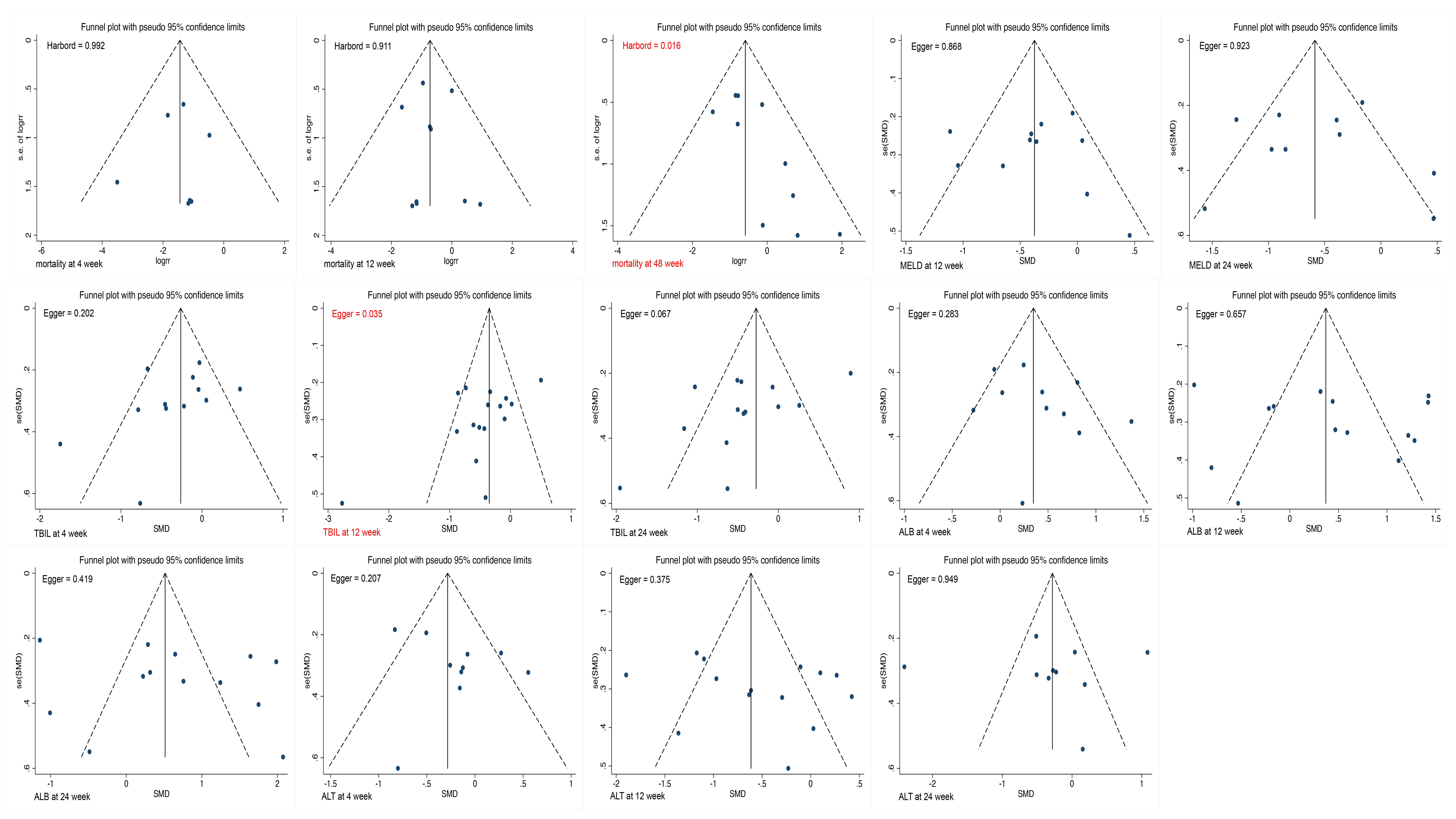

Supplement: Supplementary file 3 — Additional file 3: Figure S1. Funnel plots of mortality at weeks 4, 12, and 48; MELD at weeks 12 and 24; TBIL at weeks 4, 12 and 24; ALB at weeks 4, 12 and 24; and ALT at weeks 4, 12 and 24. Asymmetry was observed in the funnel plots of mortality at week 48 (P = 0.016) and TBIL at week 12 (P = 0.035). [file 13287_2020_1935_MOESM3_ESM.tif]

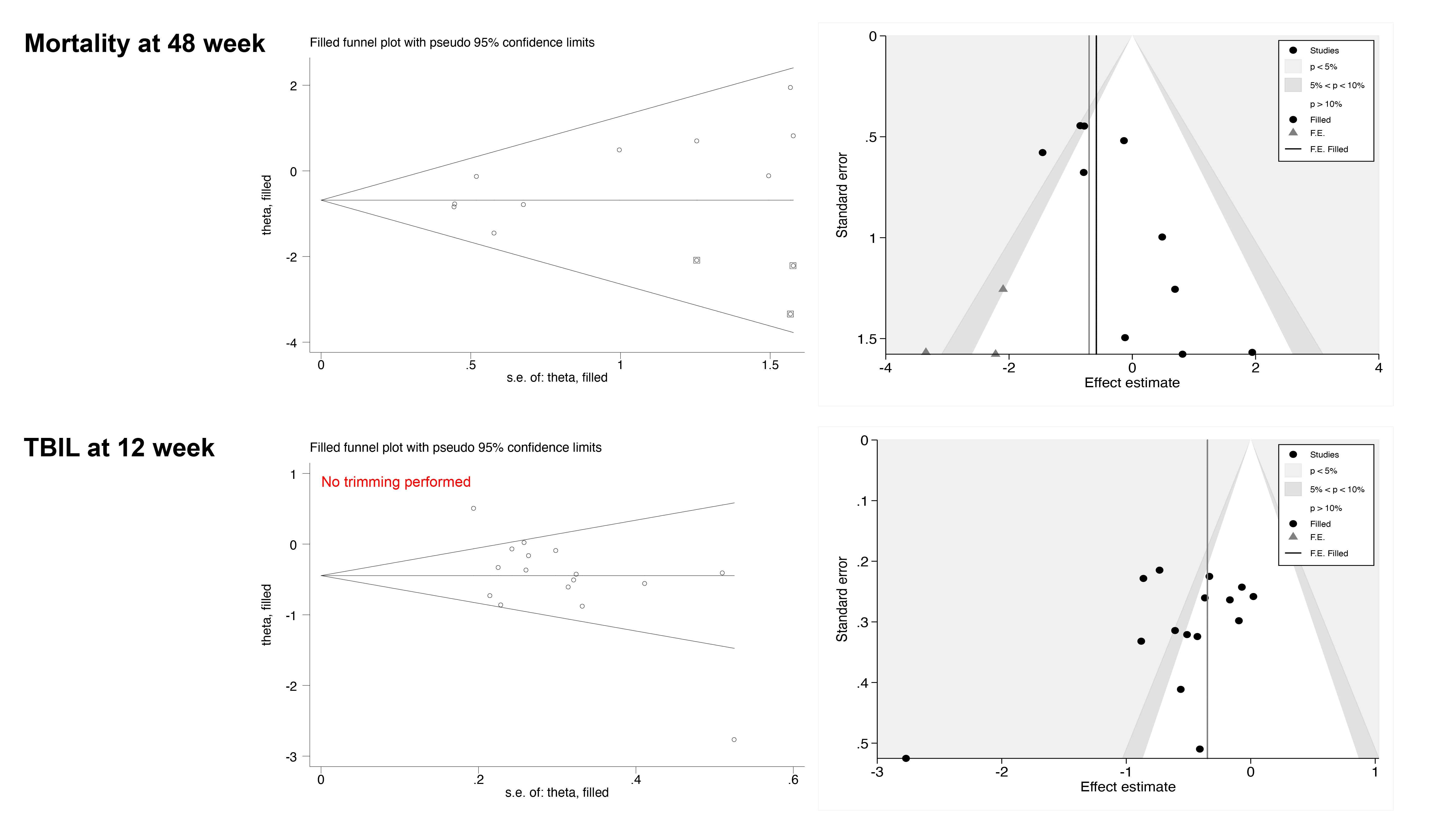

Supplement: Supplementary file 4 — Additional file 4: Figure S2. Symmetrical contour-enhanced funnel plots for mortality at week 48 and TBIL at week 12. For mortality at week 48, three hypothetical studies were filled: two plotted in the area of statistical significance and one in the area of statistical non-significance, indicating that the asymmetry in the funnel plot was partly caused by publication bias. For TBIL at week 12, no hypothetical studies were filled, indicating that the asymmetry in the funnel plot was not caused by publication bias. [file 13287_2020_1935_MOESM4_ESM.tif]
