# Supplementary material for: Therapeutic effect and safety of stem cell therapy for chronic liver disease: a systematic review and meta-analysis of randomized controlled trials
Source: Stem Cell Res Ther. 2020 Sep 25;11:419. doi: 10.1186/s13287-020-01935-w (PMC7519526; doi:10.1186/s13287-020-01935-w)
Supplement: Supplementary file 5 — Additional file 5: Table S3. Adverse events associated with stem cell therapy. [file 13287_2020_1935_MOESM5_ESM.docx]

**Additional file 5: Table S3.** Adverse events associated with stem cell therapy

| **Study** | **Side effect** | **Prognosis** |
| --- | --- | --- |
| Lyra AC et al. 2010 | Five patients complained of mild pain at the sites of bone marrow puncture and/or arterial puncture; four patients developed ecchymosis/hematoma at the site of the arterial puncture. | Resolved spontaneously |
| Salama H et al. 2010 | Mild pain and discomfort at the site of cell infusion; short term fever developed in 15 patients; mild bone pain developed in 23 patients. | Resolved spontaneously |
| Amer ME et al. 2011 | Fever was observed within 24h after injection in 10 patients; three patients showed transient shivering. | Resolved spontaneously |
| Shi M et al. 2012 | Two patients developed a self-limiting fever (37– 38°C). | Resolved spontaneously |
| Zhang YF et al. 2012 | Two patients developed a fever. | Resolved spontaneously |
| Zhang Z et al. 2012 | Four patients developed a self-limiting fever (37– 38°C) | Resolved spontaneously |
| Spahr L et al. 2013 | One patient developed a transient hematoma at the site of femoral artery puncture | Unclear |
| Xu L et al. 2014 | Low-grade fever (< 38.5°C) in four patients | Resolved spontaneously |
| Li YY et al. 2015 | Two patients developed a self-limiting fever (37– 38°C) | Resolved spontaneously |
| Suk KT et al. 2016 | Five patients developed a fever, two patients developed a hematoma, one developed chest tightness and two developed constipation | Unclear |
| Lin BL et al. 2017 | Twenty-five patients developed a fever, eight patients developed a rash and seven patients developed a diarrhea | Resolved spontaneously |
| Zhang D et al. 2017 | One patient experienced local pain and three patients had a fever | Resolved spontaneously |
| Xu WX et al. 2019 | Eleven patients had a fever | Unclear |
